# Does participation in voluntary organizations protect against risky alcohol and tobacco use? Findings from the UK panel data

**DOI:** 10.1016/j.pmedr.2019.100885

**Published:** 2019-04-29

**Authors:** Maria K. Pavlova, Matthias Lühr, Maike Luhmann

**Affiliations:** a Institute of Psychology, Faculty of Social and Behavioral Sciences, University of Jena, Am Steiger 3/1, 07743 Jena, Germany; b Institute of Gerontology, Faculty of Educational and Social Sciences, University of Vechta, Driverstraße 23, 49377 Vechta, Germany; c Faculty of Psychology, Ruhr University Bochum, Universitätsstraße 150, 44801 Bochum, Germany

**Keywords:** Age groups, Alcohol drinking, Community participation, Political activism, Smoking, Social participation

## Abstract

Drawing on the literature that posits heterogeneous influences of social networks on health behaviors, we tested whether different forms of participation in voluntary organizations predicted more or less alcohol and tobacco consumption over time. (Access preregistration at https://osf.io/guzem/) We used panel data from younger (aged 14–29 at baseline), middle-aged (aged 40–50), and older (aged 65–75) UK adults, *N*s = 1280–9073, followed from 1991 to 2014. Annual measures of smoking included status and intensity. Frequency of pub attendance was assessed biennially between 1996 and 2008. In 2010 and 2013, more precise measures of alcohol consumption were available. We conducted two-level regression analyses for the outcomes measured more than twice and residual change analyses for other outcomes. Over time (within persons), there were no significant effects on smoking. Activity in voluntary organizations predicted slightly less frequent pub attendance in younger adults. In residual change analyses, activity in voluntary organizations decreased last-week ethanol consumption and risk of heavy episodic drinking in younger women. These effects pertained mainly to service-orientated organizations. In middle-aged adults, membership and attendance at meetings of voluntary organizations predicted slightly more frequent pub attendance. Residual change analyses showed volunteering to reduce the risk of heavy episodic drinking in middle-aged men. In older adults, few significant effects emerged. Between persons, all indicators of participation were associated with less smoking, whereas membership was associated with more and activity with less frequent pub attendance. Thus, most associations between participation in voluntary organizations and substance use reflected interindividual differences.

## Highlights

• Light/moderate drinkers and nonsmokers were civically engaged much more often.
• Volunteering had isolated protective effects against heavy episodic drinking.
• Participation in service-orientated organizations had similar effects.
• There were no protective effects against smoking.
• Few age and sex differences emerged.

## 1 Introduction

Substance use habits develop, persist, and dissolve in the context of social relationships and interactions (Galea et al., 2004; Skog, 1985; Umberson et al., 2010). Consuming some psychoactive substances is seen as more socially acceptable (e.g., in Western societies, drinking alcohol; Lewis et al., 2010), whereas consuming others leads to social disapproval (e.g., smoking; Graham, 2012). Individuals who are more engaged in the society may be more likely to adjust their substance use habits to prevailing social norms (Villalonga-Olives and Kawachi, 2017).

In particular, the link between participation in voluntary organizations (which may include memberships, attendance at meetings and events, monetary donations, and volunteering) and risky substance use has long attracted the interest of youth researchers. Volunteering (i.e., unpaid voluntary work under the auspices of an organization) has been praised as a meaningful activity that gives young people a sense of maturity and responsibility and embeds them in a structured and supportive social context (Piliavin and Siegl, 2015; Youniss et al., 1999). Hence, volunteering may buffer against peer pressure, boredom, or stress, which often drive risky substance use in youth (Clark and Washington, 2011; Weybright et al., 2015). Theoretically, volunteering may activate both direct and indirect mechanisms that promote health and health behaviors (Cohen, 2004). The volunteer role contributes to better social integration, which may directly protect against risky substance use via more specific pathways of social control and engagement (Berkman et al., 2000; Cohen, 2004; Villalonga-Olives and Kawachi, 2017). Indirectly, volunteering may invoke the mechanism of social support, which is known to facilitate adaptive coping with stress and prevent turning to substance use as a coping strategy (Berkman et al., 2000; Cohen, 2004). Indeed, many cross-sectional and some longitudinal studies found protective effects of volunteering against alcohol abuse, tobacco smoking, and marijuana use in youth (Ballard et al., 2018; Pavlova et al., 2014; Theall et al., 2009; Weitzman and Chen, 2005; Youniss et al., 1999), with few exceptions (Takakura, 2015).

However, research on voluntary memberships has yielded mixed effects on substance use in youth (Bartkowski and Xu, 2007; Pavlova et al., 2014; Seid et al., 2016; Theall et al., 2009; Weitzman and Chen, 2005; Winstanley et al., 2008). Oftentimes risk-promoting effects emerged, which may be attributed to social contagion: adopting behaviors that are typical of the majority of group members (Villalonga-Olives and Kawachi, 2017). As long as no actual work is done, active memberships in voluntary organizations may provide mainly social interaction and entertainment and thus increase the likelihood of social drinking (Galea et al., 2004; Skog, 1985). A Japanese study even found a positive association between participation in youth associations and being a smoker (Takakura, 2015).

In an earlier study (Pavlova et al., 2019), we investigated whether volunteering and voluntary memberships had differential effects on alcohol and tobacco use in different age groups. A multilevel analysis of data from the German Socio-Economic Panel (SOEP) showed that most associations appeared to result from self-selection of individuals who drink moderately and do not smoke into voluntary organizations. Very few and very small effects over time emerged. Contrary to some prior findings (Ballard et al., 2018), we identified no significant differences in the effects of political and nonpolitical volunteering. As there were no significant age differences, we concluded that the past research focus on youth as an at-risk group for unhealthy substance use might have been unwarranted.

In the present study, we analyzed the data from the British Household Panel Survey (BHPS) and its continuation, Understanding Society (UndSoc). Our aim was to investigate whether different forms of participation in voluntary organizations protected from or promoted alcohol and tobacco use in the UK context. The UK has ca. 11 l of yearly alcohol consumption per capita and high prevalence rates of heavy episodic drinking (as of 2010, 35.5% in men and 20.9% in women; World Health Organization, 2014). The prevalence of current tobacco smoking is comparatively low (as of 2015, 19.9% in men and 18.4% in women; World Health Organization, 2016). Owing to anti-tobacco policies, smoking rates in the UK decreased sharply in the last decades (Office for National Statistics, 2017a). As alcohol and tobacco use in the youngest cohorts decline, while rates of consumption (of alcohol in particular) in earlier-born cohorts remain relatively high, more attention to the factors that influence substance use across adulthood becomes needed (Meng et al., 2013).

The UK is a liberal welfare state, where many social services are provided by the third sector, which relies on charitable donations and voluntary work. In the past two decades, volunteering rates have been fluctuating at about 40% (Office for National Statistics, 2017b). In the USA, volunteering is seen as a useful add-on to alcohol and drug addiction recovery programs (The Treehouse, 2018), and similar attempts have been launched in the UK, with focus group evaluations (Sheriff et al., 2014) and survey findings (Sheffield Halam University, 2015) suggesting positive effects. However, there is no rigorous research evidence from the UK on the value of volunteering in substance use prevention and treatment.

The present study focused on the role of participation in voluntary organizations in the prevention of alcohol and tobacco use in the general population. All our hypotheses referred to longitudinal effects (assess preregistration at https://osf.io/guzem/). We expected all forms of participation in voluntary organizations that did not clearly involve productive work (i.e., memberships, active participation, and attendance at meetings) to increase alcohol consumption (i.e., via the social contagion pathway (Berkman et al., 2000; Skog, 1985; Villalonga-Olives and Kawachi, 2017)). Moreover, we expected active participation to have stronger effects than a mere membership, because the former would likely involve much more social interaction. In contrast, we hypothesized that volunteering would decrease (risky) alcohol consumption (i.e., via the pathways of social support, social control, and social engagement; Berkman et al., 2000; Cohen, 2004; Villalonga-Olives and Kawachi, 2017). As smoking is often socially disapproved (Graham, 2012), we expected both active participation in voluntary organizations and volunteering to reduce its risk. However, we expected to find no effects of voluntary memberships on smoking as their potential for social control is much weaker. Furthermore, because of well-known sex differentials in substance use (World Health Organization, 2014, World Health Organization, 2016) and on the basis of our prior SOEP findings (Pavlova et al., 2019), we hypothesized that all above effects would pertain more to men than to women. We tested for age differences but expected to find none, as the only prior evidence was available from our SOEP study, where no significant age differences in longitudinal effects emerged. Finally, because prior evidence in this regard was inconclusive (Ballard et al., 2018; Pavlova et al., 2019), we explored the differences between participation in different types of voluntary organizations without hypotheses.

## 2 Methods

### 2.1 Sample and procedure

The BHPS (1991–2008) and UndSoc (2010–present, incorporated part of the BHPS samples) are annual representative surveys of British households. Each target household resident aged 16+ is eligible to be interviewed face-to-face or by telephone. We used four subsamples of the BHPS that are representative of the UK general population: Great Britain (started in 1991), Wales (1999), Scotland (1999), and Northern Ireland (2001). We did not use new subsamples from the UndSoc. Our selected subsamples included 31,553 participants surveyed at least once from 1991 to 2014. We divided participants into contrasting age groups (14–29, 40–50, and 65–75) based on their age in the year when a given outcome was assessed for the first time.

### 2.2 Measures

#### 2.2.1 Participation in voluntary organizations

Both in the BHPS and the UndSoc, two alternating sets of measures were used that were never available at the same measurement occasion. One assessed membership (“Are you currently a member of any of the kinds of organisations on this card?”) and active participation (“Whether you are a member or not, do you join in the activities of any of these organisations on a regular basis?”) in a variety of organizations. These items were administered at least biennially in the BHPS and twice in the UndSoc. We used binary indicators of membership (yes/no) and active participation (yes/no) in any organization that had civic or political agenda. For supplementary analyses, we divided all organizations into service-orientated (voluntary service groups, religious groups, and church organizations), political (political parties), and organizations that might have both political and nonpolitical agenda (e.g., environmental groups, women's institute, pensioners' groups, and professional associations).

The second set of measures assessed attendance at meetings (only BHPS) and volunteering (both BHPS and UndSoc). Between 1996 and 2008, BHPS participants reported biennially on their various leisure activities, including attending meetings of voluntary organizations and volunteering (1 = never/almost never; 5 = at least once a week). No further information on the types of organizations or activities was available here. In the UndSoc (2010, 2012, and 2014), the frequency of volunteering for any local, national, or international organization in the past 12 months was assessed on a 9-point scale (1 = on a seasonal basis; 9 = on 3 or more days a week). We added information on volunteering status (yes/no) and recoded the frequency of volunteering into a 5-point scale as in the BHPS. In supplementary analyses, we found no reliable effects of rating scale on the results reported below (see online Appendix B).

#### 2.2.2 Smoking

In almost every wave, respondents answered the question “Do you smoke cigarettes?” followed by a question for smokers only: “Approximately how many cigarettes a day do you usually smoke, including those you roll yourself?” These items had been extensively used in prior research (e.g., Booker et al., 2017; Hawkins et al., 2010). We used current smoking status (yes/no) and smoking intensity (1 = light smokers with 1–9 cigarettes per day; 2 = moderate smokers with 10–19 cigarettes per day; 3 = heavy smokers with 20+ cigarettes per day; Chiolero et al., 2006).

#### 2.2.3 Alcohol consumption

Starting in 1996, BHPS participants reported biennially on the frequency of pub attendance^1^ (“How frequently do you go for a drink at a pub or club?”) using same 5-point rating scale as for volunteering. Much more precise measures were administered only twice in the UndSoc (2010 and 2013). Participants reported how often they had consumed alcohol in the past 12 months (1 = not at all; 8 = almost every day), in the past seven days (number of days), and the maximum number of four specific drink types consumed in one day. We used information on drink types to calculate the maximum ethanol consumption (ml) in the past seven days (National Health Service, 2018) and employed cut-off values for ethanol consumption in grams (World Health Organization, 2000) to determine the risk of heavy episodic drinking in the past seven days (1 = low risk, <41 g in men or <21 g in women; 2 = medium risk, <61 g in men or <41 g in women; 3 = high risk, <101 g in men or <61 g in women; 4 = very high risk, >100 g in men or >60 g in women). The BHPS measure of pub attendance (2008) correlated at 0.28–0.30 with these more precise measures from the UndSoc (2010). Thus, it was acceptable as a proxy measure.

#### 2.2.4 Control variables

The first set of control variables included sociodemographic indicators: sample origin, sex, age, highest academic qualification, employment status, equalized disposable household income in euro (logged), occupational status, cohabiting with a partner/spouse, and underage children in the household. The second set included subjective health (one-item measure of global health; Bowling, 2005) and emotional well-being (six items from the General Health Questionnaire; Goldberg et al., 1997). The third set included other leisure activities: self-reported frequencies of church attendance, going out, socializing, doing sports, and manual work. Item wordings are available in the online preregistration form: https://osf.io/guzem/.

### 2.3 Statistical analyses

We conducted all analyses in the three age groups separately. To disentangle interindividual differences (between-person effects) from intraindividual change (within-person effects), we conducted multilevel regression analyses (Fisher et al., 2018; Wang and Maxwell, 2015) for the outcomes measured at least three times. For the first set of predictors (membership and active participation in voluntary organizations), we tested three models: (1) adjusted only for age at the within level, (2) adjusted for age (within level) and sociodemographic indicators (both levels), (3) adjusted for age (within level) and health and well-being (both levels). For the second set of predictors (attendance at meetings and volunteering), a fourth model controlled for other leisure activities at the within level. As precise measures of alcohol consumption were available at only two waves, we conducted single-level residual change analyses with these measures, whereby membership, active participation in voluntary organizations, and volunteering were central predictors. All analyses were conducted in MPlus 8.0 (Muthén and Muthén, 2017). A full description of our analytical approach can be accessed via the online preregistration at https://osf.io/guzem/.

## 3 Results

Descriptive statistics are shown in Table 1.

**Table 1:** Descriptive statistics for the key study variables.

Variables	*M* (*SD*)	%	*n* persons	*n* observations	*M* (*SD*)	%	*n* persons	*n* observations	*M* (*SD*)	%	*n* persons	*n* observations
*For multilevel analyses of pub attendance*	Age 14–29 (1996)				Age 40–50 (1996)				Age 65–75 (1996)			
Attendance at meetings (1–5)	1.46 (1.06)	–	6614	20,748	1.71 (1.25)	–	3454	13,670	1.90 (1.46)	–	1842	7018
Volunteering (1–5)	1.41 (1.04)	–	7008	28,548	1.60 (1.26)	–	3547	18,712	1.56 (1.27)	–	1857	8487
Membership	–	34.1	8368	36,655	–	49.3	4191	27,168	–	42.0	2382	14,015
Active participation	–	19.9	8365	36,541	–	31.1	4191	27,081	–	35.0	2385	13,994
Pub attendance (1–5)	3.89 (1.20)	–	6801	20,748	3.20 (1.47)	–	3484	15,900	2.11 (1.56)	–	1844	7902

*For multilevel analyses of smoking*	Age 14–29 (1992)				Age 40–50 (1992)				Age 65–75 (1992)			
Attendance at meetings (1–5)	1.51 (1.08)	–	6654	22,658	1.75 (1.30)	–	3352	13,352	1.90 (1.48)	–	1468	5352
Volunteering (1–5)	1.43 (1.07)	–	6942	31,136	1.64 (1.31)	–	3437	18,356	1.51 (1.23)	–	1477	6113
Membership	–	38.2	8556	42,785	–	48.6	4060	26,662	–	43.0	2018	11,152
Active participation	–	22.4	8553	42,634	–	31.5	4061	26,592	–	36.2	2021	11,129
Smoker	–	31.4	9073	72,892	–	25.2	4141	44,329	–	14.1	2035	17,491
Smoking intensity			4009	22,660			1469	11,107			472	2451
Light smokers	–	23.6			–	14.3			–	32.4		
Moderate smokers	–	42.5			–	33.9			–	45.6		
Heavy smokers	–	33.9			–	51.8			–	22.0		

*For two-wave analyses of alcohol consumption*	Age 14–29 (2010)				Age 40–50 (2010)				Age 65–75 (2010)			
Volunteering (1–9) _2010_	1.72 (1.85)	–	2355	–	1.82 (2.00)	–	2421	–	2.19 (2.40)	–	1487	–

Membership _2011_	–	24.0	1768	–	–	44.9	2114	–	–	45.1	1348	–
Active participation _2011_	–	19.7	1768	–	–	31.7	2114	–	–	38.2	1345	–
Frequency of alcohol consumption in past 12 months (1–8) _2010_	4.29 (1.44)	–	2046	–	4.75 (1.83)	–	2154	–	4.33 (2.21)	–	1280	–
Frequency of alcohol consumption in past 12 months (1–8) _2013_	4.09 (1.62)	–	1368	–	4.55 (1.96)	–	1729	–	4.14 (2.30)	–	1001	–
Maximum ethanol consumption (ml) past 7 days _2010_	77.97 (99.32)	–	1994	–	60.32 (66.61)	–	2105	–	34.51 (45.16)	–	1230	–
Maximum ethanol consumption (ml) past 7 days _2013_	66.30 (85.28)	–	1368	–	57.00 (72.47)	–	1727	–	30.27 (50.73)	–	1000	–
Risk of heavy episodic drinking _2010_			1994	–			2105	–			1230	–
Low risk	–	47.0			–	43.3			–	64.1		
Medium risk	–	8.3			–	14.1			–	16.3		
High risk	–	11.6			–	18.7			–	11.6		
Very high risk	–	33.1			–	23.9			–	7.9		
Risk of heavy episodic drinking _2013_			1368	–			1727	–			1000	–
Low risk	–	51.5			–	47.7			–	69.0		
Medium risk	–	8.4			–	13.0			–	14.5		
High risk	–	12.5			–	17.0			–	10.7		
Very high risk	–	27.6			–	22.4			–	5.8		

Note. Summary statistics across persons and observations are shown. *n* = number of valid cases. Dash = not applicable. Age groups were defined according to participants' age in a given year (shown in parentheses). The data come from UK panel surveys conducted in 1991–2014.

### 3.1 Smoking: findings from multilevel analyses

#### 3.1.1 Within-person effects

Effect sizes for the predictors of interest are summarized in Tables 2 (unadjusted models, within level) and 3 (unadjusted models, between level), whereas online Appendix A shows full results from multilevel regression analyses. (The formulae for calculating probability differences are presented in online Appendix E.) Across age groups, the probability of being a smoker and smoking intensity generally decreased over time (see online Appendix A), which was in line with prior research (Yong et al., 2012). However, we found no significant effects of occasion-specific membership, active participation, attendance at meetings, or volunteering on smoking status or intensity in any age group (see Table 2). Therefore, we did not test for age differences. Separate analyses by sex yielded some significant effects of attendance at meetings and volunteering on the lower smoking probability in women (see online Appendix C). However, they did not hold in all models with control variables, and sex differences were not significant (at *p* < .01).

**Table 2:** Summary of findings from multilevel analyses: within-person effects.

Predictors and outcomes	Younger adults		Middle-aged adults		Older adults	
	% change	95% CI	% change	95% CI	% change	95% CI
Outcome: Pub attendance _t_						
1st set of predictors						
Membership in organizations _*t*−1_	−2.7	[−5.8, 0.1]	4.0	[0.1, 8.0]	−3.6	[−11.1, 4.7]
Active participation in organizations _*t*−1_	−5.6	[−8.5, −2.3]	−3.4	[−7.1, 0.4]	3.6	[−4.4, 10.8]
2nd set of predictors						
Attendance at meetings _*t*_	−0.6	[−1.7, 0.3]	2.2	[0.9, 3.6]	1.2	[−1.2, 3.6]
Volunteering _*t*_	0.9	[−0.2, 2.0]	−1.4	[−2.8, 0.1]	1.8	[−0.7, 4.4]

Outcome: Smoker _t_						
1st set of predictors						
Membership in organizations _*t*−1_	0.2	[−1.5, 2.0]	−0.7	[−2.7, 1.2]	−1.2	[−7.7, 5.6]
Active participation in organizations _*t*−1_	0.0	[−0.1, 0.0]	−1.3	[−3.5, 0.9]	−4.2	[−11.5, 1.6]
2nd set of predictors						
Attendance at meetings _*t*_	−0.9	[−1.9, 0.4]	−0.4	[−1.9, 0.1]	−0.6	[−4.0, 3.0]
Volunteering _*t*_	0.1	[−1.0, 1.1]	0.1	[−1.3, 1.6]	−3.3	[−6.8, 0.5]

Outcome: Smoking intensity _t_						
1st set of predictors						
Membership in organizations _*t*−1_	−0.2	[−4.2, 3.7]	1.6	[−1.8, 5.4]	−4.9	[−18.6, 9.6]
Active participation in organizations _*t*−1_	0.2	[−5.2, 4.8]	−1.8	[−6.2, 2.3]	−6.7	[−23.3, 9.3]
2nd set of predictors						
Attendance at meetings _*t*_	−0.6	[−2.8, 1.5]	−1.0	[−3.2, 1.0]	1.5	[−5.4, 8.2]
Volunteering _*t*_	−0.6	[−2.7, 1.4]	−1.3	[−3.5, 0.9]	−3.1	[−10.1, 4.5]

Note. Effects from the models adjusted only for age at the within level are shown. CI = Bayesian credibility interval. The data come from UK panel surveys conducted in 1991–2014.

#### 3.1.2 Between-person effects

All indicators of participation were significantly associated with no or less smoking on the between-person level (in every age group and in almost all models; see Table 3 and online Appendix A). Effect sizes for significant associations ranged from 7% to 42% difference for smoking status and from 12% to 41% difference for smoking intensity (i.e., in the probability to be a heavy smoker).

**Table 3:** Summary of findings from multilevel analyses: between-person effects.

Predictors and outcomes	Younger adults		Middle-aged adults		Older adults	
	% difference	95% CI	% difference	95% CI	% difference	95% CI
Outcome: Pub attendance across obs.						
1st set of predictors						
Ever member in organizations	10.9	[5.6, 16.4]	12.8	[2.0, 24.5]	37.5	[12.7, 64.0]
Ever active in organizations	−25.4	[−29.9, −20.8]	−22.8	[−31.1, −14.3]	−41.2	[−54.8, −25.4]
2nd set of predictors						
Attendance at meetings across obs.	−5.0	[−7.9, −2.2]	−5.6	[−11.1, −0.2]	−5.0	[−13.4, 3.9]
Volunteering across obs.	0.1	[−2.8, 3.1]	−5.3	[−10.6, 0.4]	3.9	[−4.8, 12.7]

Outcome: Smoker across obs.						
1st set of predictors						
Ever member in organizations	−27.8	[−32.8, −22.9]	−31.7	[−39.6, −23.7]	−27.8	[−44.6, −10.0]
Ever active in organizations	−20.1	[−25.4, −14.8]	−28.0	[−35.4, −20.1]	−41.8	[−55.5, −26.5]
2nd set of predictors						
Attendance at meetings across obs.	−19.9	[−24.1, −16.0]	−23.3	[−30.8, −15.6]	−31.5	[−43.7, −18.3]
Volunteering across obs.	−7.0	[−11.2, −2.8]	−23.0	[−30.5, −15.4]	−19.4	[−32.6, −4.5]

Outcome: Smoking intensity across obs.						
1st set of predictors						
Ever member in organizations	−22.5	[−29.3, −15.4]	−7.4	[−16.3, 0.8]	−1.5	[−28.8, 29.1]
Ever active in organizations	−11.9	[−19.4, −4.3]	−16.0	[−24.8, −7.1]	−40.5	[−60.4, −15.5]
2nd set of predictors						
Attendance at meetings across obs.	−4.7	[−9.5, 0.2]	−13.4	[−19.7, −6.9]	−9.0	[−24.2, 7.6]
Volunteering across obs.	−3.0	[−7.7, 2.3]	0.3	[−6.3, 6.7]	−4.5	[−19.6, 11.6]

Note. Effects from the models adjusted only for age at the within level are shown. CI = Bayesian credibility interval. The data come from UK panel surveys conducted in 1991–2014.

### 3.2 Pub attendance: findings from multilevel analyses

#### 3.2.1 Within-person effects

In concordance with prior research (Knott et al., 2017), across age groups, the frequency of pub attendance decreased over time (see online Appendix A). In unadjusted models, being a member of a voluntary organization significantly predicted a 4% higher probability of weekly pub attendance in the next year in middle-aged adults (see Table 2). Although this effect was no longer significantly different from zero in the models with control variables (see online Appendix A), it was significantly (*p* < .01) more positive than the respective effect in younger adults. In the latter, being active in a voluntary organization significantly predicted a 6% lower probability of weekly pub attendance in the next year across all models. However, in none of the age groups did we find significant differences (at *p* < .01) between the effects of membership and active participation in voluntary organizations.

Regarding the second set of predictors, in middle-aged adults, a 1*SD* higher occasion-specific attendance at meetings was significantly associated with a 2% higher probability of weekly pub attendance at the same measurement occasion (see Table 2; this effect held across all models with control variables). This effect was significantly (*p* < .01) more positive than the association between volunteering and pub attendance in the same age group. Moreover, it was significantly more positive than the same effect in younger adults.

In separate analyses by sex, we found that most significant effects reported in this section pertained to women (see online Appendix C). However, there were no significant sex differences (at *p* < .01).

#### 3.2.2 Between-person effects

Across age groups, participants who had ever been a member of a voluntary organization attended pubs significantly more often (11–38% difference for weekly pub attendance; see Table 3). In contrast, persons who had ever been active in a voluntary organization attended pubs significantly less often (23–41% difference). A 1*SD* more frequent attendance at meetings was associated with ca. 5% lower probability of weekly pub attendance in younger and middle-aged adults. These between-person effects were sometimes accounted for by control variables (see online Appendix A). Associations with the frequency of volunteering were not significant.

### 3.3 Alcohol consumption: findings from two-wave analyses

In younger adults, active participation in voluntary organizations in 2011 significantly decreased the last-week risk of heavy episodic drinking from 2010 to 2013 (OR = 0.67). In contrast, in middle-aged adults, membership of a voluntary organization in 2011 significantly increased this risk (OR = 1.34). The latter effect was explained by sociodemographic control variables. Age differences were not significant (at *p* < .01), and there were no significant effects on other indicators of alcohol consumption (see online Appendix A).

More significant effects emerged in separate analyses by sex (see online Appendix C). In younger women, active participation in a voluntary organization in 2011 significantly predicted a residual decrease in last-week ethanol consumption from 2010 to 2013 (ß = −0.33). This effect differed significantly (*p* < .01) from the respective effect in younger men (ß = 0.14, *ns*). Likewise, active participation in a voluntary organization in 2011 significantly decreased the last-week risk of heavy episodic drinking from 2010 to 2013 in younger women (OR = 0.55) but not in younger men (OR = 0.95). However, sex difference was not significant here. In middle-aged men, both linear and quadratic effects of volunteering in 2010 on the last-week risk of heavy episodic drinking in 2013 were significant. Overall, volunteering predicted a decreasing risk, but this effect was more pronounced at lower and less pronounced at higher levels of volunteering (for a change in volunteering from −1*SD* to *M*: OR = 0.41; for a change in volunteering from *M* to +1*SD*: OR = 0.71). The respective effects were not significant in middle-aged women, but sex differences did not reach significance (at *p* < .01).

### 3.4 Supplementary analyses

We explored whether the effects of membership and active participation varied across types of organizations: political parties, service-orientated organizations, and organizations with mixed political and nonpolitical agenda (see Table 4 and online Appendix D). Findings indicated that membership or activity in service-orientated organizations decreased pub attendance in younger and older adults (multilevel analyses) and the risk of heavy episodic drinking in younger adults (two-wave analyses). In contrast, membership or activity in a political party predicted higher alcohol consumption in younger and middle-aged adults (two-wave analyses). Results for organizations with mixed agenda were less consistent, with both promotive (risk of heavy episodic drinking and smoking intensity, the latter not shown in Table 4) and protective (pub attendance) effects (which were not significant in all models) found in middle-aged adults.

**Table 4:** Summary of central findings for different types of organizations: longitudinal effects on alcohol consumption.

Predictors and outcomes	Younger adults		Middle-aged adults		Older adults	
Outcome: Pub attendance _*t*_^a^	% change	95% CI	% change	95% CI	% change	95% CI
Membership in service-orientated organizations _*t*−1_	−2.2	[−8.2, 3.7]	−2.3	[−8.8, 4.3]	−17.6	[−27.5, −6.5]
Active participation in service-orientated organizations _*t*−1_	−7.5	[−12.7, −2.3]	3.2	[−3.7, 9.8]	6.3	[−5.4, 18.4]
Membership in political parties _*t*−1_	13.3	[−5.2, 32.2]	−1.7	[−17.7, 14.1]	4.7	[−15.0, 26.0]
Active participation in political parties _*t*−1_	−4.5	[−25.8, 19.5]	3.2	[−3.7, 9.8]	14.2	[−8.4, 40.0]
Membership in organizations with mixed agenda _*t*−1_	−2.7	[−5.8, 0.1]	3.6	[−0.8, 7.8]	3.2	[−5.8, 12.7]
Active participation in organizations with mixed agenda _*t*−1_	−2.2	[−5.5, 1.2]	−4.2	[−8.8, −0.2]	4.5	[−4.7, 14.3]

Outcome: Alcohol consumption past 12 months _2013_^b^	β	95% CI	β	95% CI	β	95% CI
Membership in service-orientated organizations _*2011*_	0.12	[−0.09, 0.32]	0.00	[−0.14, 0.14]	−0.03	[−0.16, 0.11]
Active participation in service-orientated organizations _*2011*_	−0.11	[−0.32, 0.10]	0.00	[−0.13, 0.14]	0.10	[−0.05, 0.24]
Membership in political parties _*2011*_	−0.07	[−0.65, 0.51]	−0.03	[−0.35, 0.30]	0.14	[−0.07, 0.34]
Active participation in political parties _*2011*_	0.92	[0.22, 1.62]	0.00	[−0.33, 0.33]	0.03	[−0.21, 0.27]
Membership in organizations with mixed agenda _*2011*_	−0.07	[−0.21, 0.08]	0.06	[−0.01, 0.13]	−0.02	[−0.13, 0.09]
Active participation in organizations with mixed agenda _*2011*_	0.10	[−0.07, 0.26]	0.00	[−0.08, 0.07]	0.00	[−0.13, 0.12]

Outcome: Maximum ethanol consumption past 7 days _2013_^b^	β	95% CI	β	95% CI	β	95% CI
Membership in service-orientated organizations _*2011*_	0.04	[−0.20, 0.29]	0.03	[−0.15, 0.21]	0.00	[−0.17, 0.17]
Active participation in service-orientated organizations _*2011*_	−0.23	[−0.47, 0.00]	−0.12	[−0.29, 0.05]	0.15	[−0.03, 0.32]
Membership in political parties _*2011*_	−0.28	[−0.77, 0.21]	0.29	[0.05, 0.54]	0.09	[−0.32, 0.50]
Active participation in political parties _*2011*_	0.83	[0.24, 1.43]	0.08	[−0.36, 0.52]	0.15	[−0.44, 0.73]
Membership in organizations with mixed agenda _*2011*_	−0.03	[−0.18, 0.13]	0.08	[−0.01, 0.17]	0.06	[−0.08, 0.20]
Active participation in organizations with mixed agenda _*2011*_	0.03	[−0.18, 0.23]	0.10	[0.00, 0.20]	−0.13	[−0.28, 0.02]

Outcome: Risk of heavy episodic drinking past 7 days _2013_^b^	Exp (B)	95% CI	Exp (B)	95% CI	Exp (B)	95% CI
Membership in service-orientated organizations _*2011*_	1.04	[0.64, 1.69]	1.04	[0.68, 1.60]	1.29	[0.75, 2.20]
Active participation in service-orientated organizations _*2011*_	0.61	[0.39, 0.97]	0.86	[0.57, 1.31]	1.26	[0.70, 2.26]
Membership in political parties _*2011*_	0.52	[0.14, 2.03]	1.61	[0.64, 4.05]	1.74	[0.52, 5.80]
Active participation in political parties _*2011*_	4.67	[0.84, 26.17]	0.72	[0.24, 2.15]	0.54	[0.08, 3.89]
Membership in organizations with mixed agenda _*2011*_	1.08	[0.80, 1.47]	1.23	[1.04, 1.62]	1.19	[0.78, 1.80]
Active participation in organizations with mixed agenda _*2011*_	0.97	[0.65, 1.44]	1.05	[0.81, 1.36]	1.01	[0.64, 1.58]

Note. CI = Bayesian credibility interval. The data come from UK panel surveys conducted in 1991–2014.

a Findings from multilevel analyses (within level, controlled only for age).

b Findings from residual change analyses (controlled for the lagged outcome).

Additionally, we checked whether the significant age differences in the effects of voluntary memberships and attendance at meetings on pub attendance might be due to cohort effects. When we defined age groups on the basis of the participants' age in 2004 (rather than 1996), the effects of participation indicators became more negative in younger adults and changed little in middle-aged adults. Age differences grew even stronger. These results speak for cohort effects, whereby the effects of participation indicators on pub attendance become increasingly more negative in younger cohorts.

## 4 Discussion

Findings of this study based on large-scale UK panel data qualify the widespread assumption that volunteering may be helpful in substance use prevention. We did sometimes find participation in voluntary organizations to predict less risky alcohol consumption over time, but these effects were mostly small, circumscribed to particular types of organizations (i.e., service-orientated), and were only in some cases attributable to volunteering, whereas in other cases, a mere membership in the “right” type of organization sufficed. Moreover, there were virtually no longitudinal effects of participation in voluntary organizations on smoking. In contrast, sizable associations between average patterns of substance use and participation in voluntary organizations across observations emerged, suggesting a self-selection of light and moderate drinkers and nonsmokers into voluntary organizations (cf. Pavlova et al., 2019).

Our expectation that most forms of participation in voluntary organizations would foster alcohol use (via social drinking) whereas volunteering (as a meaningful productive activity) would be protective received some support only in middle-aged adults. In contrast, most effects of participation in voluntary organizations (not limited to volunteering) in younger adults could be seen as protective. These differences between younger and middle-aged adults could be attributable to cohort effects. In the UK, younger cohorts show greater rates of abstinence, which, in turn, have been linked to a growing prevalence of young first- and second-generation immigrants from high-abstinence cultures (Meng et al., 2013). As civic engagement of migrants is often connected to their cultural and religious traditions (Jensen, 2010), it may be protective against alcohol use. In supplementary analyses, we found indications that it was membership or activity in service-orientated organizations (which included religious associations) that predicted lower alcohol consumption in younger adults. Moreover, individuals with a migration background were more likely to engage in these organizations and were also overrepresented in the youngest group.

We found few significant longitudinal effects in older adults (although no significant differences from other age groups emerged either). Nevertheless, there was a robust negative effect of being a member of a service-orientated organization on pub attendance at the next measurement occasion, which may be understood as protective. Together with our results for younger adults, this finding may point at the importance of organizational ethos (i.e., social control; Berkman et al., 2000), which, in turn, may be related to religious roots of many such organizations.

In striking contrast to service-orientated organizations, membership or activity in a political party predicted higher alcohol consumption in younger and middle-aged adults. This post-hoc finding that emerged only in residual change analyses should be treated with caution. However, it aligns well with anecdotal knowledge that social drinking and drinking excesses have long been part of male-dominated political culture in the UK and elsewhere (Wright, 2017). Actually, we expected all longitudinal effects of participation in voluntary organizations on alcohol and tobacco use to be stronger in men than in women. This expectation was not corroborated by findings, with significant effects on alcohol use sometimes found only in men, only in women, or in both sexes, and hardly any significant sex differences. The UK has been moving to less gendered drinking patterns in the last decades, and the factors promoting or protecting against risky alcohol use may have become more uniform (2013).

At the level of interindividual differences, our findings were largely consistent with our earlier results from a German panel study (Pavlova et al., 2019): Individuals who participated in voluntary organizations more (across observations) were less likely to smoke, whereas voluntary memberships in particular were associated with a greater frequency of pub attendance across observations. These associations suggest that individuals with a socially desirable pattern of substance use (no smoking and some social drinking) may self-select into voluntary organizations. A robust negative association between various kinds of activity in voluntary organizations and pub attendance across observations might also be attributed to self-selection (e.g., more conscientious or more religious individuals are likelier to work for voluntary organizations and probably attend pubs less often). Thus, some prior studies that could not disentangle interindividual differences from intraindividual change might have overestimated the protective effects of volunteering. The same limitation pertains to single-level residual change analyses in the present study, where the longitudinal effects of volunteering and other types of participation in voluntary organizations appeared sizable.

This study had other limitations common to large panel studies: self-report measures, differences in instruments across waves and especially between the BHPS and UndSoc, predefined intervals between waves, and longitudinal attrition. Some of these were partly addressed in our statistical analyses. Moreover, only pub attendance was available in multilevel analyses as a proxy measure of alcohol use. This measure showed satisfactory correlations (about 0.3) with more precise indicators assessed two years later. Although a fine differentiation among types of voluntary organizations was made, information on specific activities and responsibilities within organizations was lacking. On the positive side, we were able to distinguish among levels of involvement in voluntary organizations.

## 5 Conclusions

Our findings from two nationally representative UK panel studies confirm that individuals' participation in voluntary organizations is related to their levels of alcohol and tobacco use. However, the greater part of such associations reflects interindividual differences. We found some evidence for modest protective effects of either volunteering or any kind of participation in service-orientated organizations against alcohol use but not against smoking. These protective effects may be specific to the UK context and may have to do with social norms prevalent in service-orientated organizations that often have religious underpinnings.

## Appendix Supplementary data

Supplementary material Image 1

## Footnotes

1 Among underage participants (aged 14–17 at the time of DV measurement), 67% reported some pub attendance, with 26.5% reporting to go for a drink at least once a week. Likewise, 26.1% of underage participants were classified as having a very high risk of heavy episodic drinking. Even though these figures were smaller than among those aged 18–29, they warranted the inclusion of underage participants into the younger age group.
