# Supplementary material for: Does participation in voluntary organizations protect against risky alcohol and tobacco use? Findings from the UK panel data
Source: Prev Med Rep. 2019 Apr 29;14:100885. doi: 10.1016/j.pmedr.2019.100885 (PMC6517570; doi:10.1016/j.pmedr.2019.100885)
Supplement: Supplementary file 1 — Supplementary material [file mmc1.docx]

**Online Supplement**

**Appendix A** Full regression results (with the effects of control variables)

**Appendix B** Effects of the rating scale of volunteering on smoking

**Appendix C** Separate analyses by sex: Compact regression results (without the effects of control variables)

**Appendix D** Supplementary analyses of different types of organizations: Compact regression results (without the effects of control variables)

**Appendix E** The calculation of effect sizes

**Appendix A**

*Effects of Membership and Active Participation in Voluntary Organizations on Pub Attendance and Smoking in Younger Adults: Full Results*

| Predictors | Pub attendance *_t_* | | | Smoker *_t_* | | | Smoking intensity *_t_* | | |
| --- | --- | --- | --- | --- | --- | --- | --- | --- | --- |
|  | Model 1 | Model 2 | Model 3 | Model 1 | Model 2 | Model 3 | Model 1 | Model 2 | Model 3 |
| *Within* |  |  |  |  |  |  |  |  |  |
| Membership *_t-1_* | -0.052  [-0.111, 0.002] | -0.065*  [-0.118, -0.006] | -0.060  [-0.118, 0.005] | 0.010  [-0.063, 0.083] | -0.004  [-0.072, 0.070] | 0.009  [-0.064, 0.082] | -0.003  [-0.080, 0.071] | -0.006  [-0.085, 0.069] | -0.001  [-0.074, 0.072] |
| Active participation *_t-1_* | -0.107**  [-0.163, -0.061] | -0.087**  [-0.147, -0.032] | -0.105**  [-0.160, -0.052] | -0.008  [-0.095, 0.063] | 0.003  [-0.083, 0.084] | -0.013  [-0.094, 0.074] | 0.004  [-0.101, 0.091] | 0.009  [-0.079, 0.101] | 0.009  [-0.082, 0.093] |
| Age *_t_* | -0.066**  [-0.071, -0.061] | -0.043**  [-0.048, -0.037] | -0.066**  [-0.070, -0.061] | -0.054**  [-0.057, -0.050] | -0.049**  [-0.053, -0.045] | -0.054**  [-0.058, -0.050] | -0.003  [-0.008, 0.001] | -0.003  [-0.008, 0.001] | -0.004  [-0.008, 0.001] |
| Unemployed *_t_* |  | -0.204**  [-0.308, -0.099] |  |  | -0.093  [-0.208, 0.019] |  |  | -0.176**  [-0.275, -0.079] |  |
| Out of the labor market *_t_* |  | -0.412**  [-0.475, -0.352] |  |  | -0.080*  [-0.154, -0.008] |  |  | 0.003  [-0.072, 0.074] |  |
| Income (logged) *_t_* |  | 0.002  [-0.029, 0.033] |  |  | -0.023  [-0.059, 0.010] |  |  | -0.021  [-0.057, 0.013] |  |
| Cohabiting with partner *_t_* |  | -0.692**  [-0.743, -0.641] |  |  | -0.105**  [-0.162, -0.042] |  |  | -0.053  [-0.113, 0.008] |  |
| Children in the household *_t_* |  | -0.786**  [-0.836, -0.739] |  |  | -0.380**  [-0.430, -0.321] |  |  | -0.047  [-0.109, 0.014] |  |
| Subjective health^a^ *_t-1_* |  |  | 0.000  [-0.006, 0.005] |  |  | -0.005  [-0.011, 0.000] |  |  | 0.003  [-0.002, 0.009] |
| Emotional well-being  *_t-1_* |  |  | -0.054*  [-0.101, -0.009] |  |  | -0.136**  [-0.184, -0.089] |  |  | 0.002  [-0.048, 0.051] |
| R² | 0.085 | 0.326 | 0.085 | 0.061 | 0.102 | 0.065 | 0.001 | 0.005 | 0.001 |
| *Between* |  |  |  |  |  |  |  |  |  |
| Ever member | 0.208**  [0.107, 0.311] | 0.006  [-0.085, 0.099] | 0.149**  [0.050, 0.247] | -1.193**  [-1.424, -0.976] | -0.597**  [-0.811, -0.377] | -1.076**  [-1.309, -0.847] | -0.451**  [-0.601, -0.304] | -0.147*  [-0.300, 0.000] | -0.392**  [-0.535, -0.246] |
| Ever active | -0.503**  [-0.599, -0.409] | -0.234**  [-0.322, -0.146] | -0.508**  [-0.600, -0.410] | -0.852**  [-1.084, -0.622] | -0.558**  [-0.780, -0.334] | -0.848**  [-1.076. -0.626] | -0.232**  [-0.387, -0.083] | -0.101  [-0.251, 0.047] | -0.233**  [-0.382, -0.079] |
| Scotland extension sample |  | -0.467**  [-0.571, -0.363] |  |  | 0.120  [-0.141, 0.388] |  |  |  |  |
| Wales extension sample |  | -0.207**  [-0.314, -0.094] |  |  | -0.069  [-0.351, 0.220] |  |  |  |  |
| Northern Ireland extension sample |  | -0.699  [-0.800, -0.592] |  |  | -0.126  [-0.388, 0.137] |  |  |  |  |
| Woman |  | -0.258  [-0.326, -0.177] |  |  | -0.303**  [-0.479, -0.119] |  |  |  |  |
| Average highest academic qualification |  | 0.122**  [0.092, 0.153] |  |  | -0.424**  [-0.495, -0.350] |  |  |  |  |
| Average occupational status |  | 0.021  [-0.020, 0.062] |  |  | -0.288**  [-0.402, -0.181] |  |  |  |  |
| Average income |  | 0.096**  [0.028, 0.164] |  |  | -0.650**  [-0.811, -0.482] |  |  |  |  |
| Average subjective health |  |  | 0.061  [0.050, 0.072] |  |  | -0.181**  [-0.205. -0.157] |  |  | -0.082**  [-0.099, -.065] |
| Average emotional well-being |  |  | 0.080  [-0.038, 0.197] |  |  | -0.635**  [-0.905, -0.384] |  |  | -0.109**  [-0.274, 0.059] |
| Threshold 1 | -2.587**  [-2.653, -2.538] | -3.803**  [-3.875, -3.655] | -2.641**  [-2.706, -2.581] | 0.147**  [0.110, 0.185] | 0.609**  [0.422, 0.800] | 0.606**  [0.470, 0.746] | -1.413**  [-1.519, -1.337] | -1.333**  [-1.457, -1.180] | -1.371**  [-1.480, -1.300] |
| Threshold 2 | -2.146**  [-2.205, -2.098] | -3.356**  [-3.432, -3.205] | -2.200**  [-2.262, -2.140] | N/A | N/A | N/A | 0.775**  [0.669, 0.846] | 0.857**  [0.745, 1.019] | 0.815**  [0.723, 0.891] |
| Threshold 3 | -1.085**  [-1.138, -1.035] | -2.256**  [-2.324. -2.121] | -1.135**  [-1.197, -1.082] | N/A | N/A | N/A | N/A | N/A | N/A |
| Threshold 4 | 0.245  [0.192, 0.298] | -0.851**  [-0.912, -0.732] | 0.198**  [0.141, 0.245] | N/A | N/A | N/A | N/A | N/A | N/A |
| Residual variance (σ^2^*_u_*) | 1.908**  [1.803, 2.024] | 1.388**  [1.302, 1.480] | 1.851**  [1.740, 1.961] | 12.569**  [11.586, 13.462] | 11.317**  [10.482, 12.405] | 11.824**  [10.919, 12.714] | 3.073**  [2.849, 3.310] | 2.752**  [2.553, 2.970] | 2.944  [2.739, 3.156] |
| R² | 0.022 | 0.091 | 0.060 | 0.062 | 0.145 | 0.121 | 0.029 | 0.148 | 0.072 |

*Note.* Cells represent unstandardized probit regression coefficients with Bayesian credibility intervals in square brackets.

^a^ Rescaled into quantile ranks.

* *p* < .05. ** *p* < .01.

*Effects of Membership and Active Participation in Voluntary Organizations on Pub Attendance and Smoking in Middle-Aged Adults: Full Results*

| Predictors | Pub attendance *_t_* | | | Smoker *_t_* | | | Smoking intensity *_t_* | | |
| --- | --- | --- | --- | --- | --- | --- | --- | --- | --- |
|  | Model 1 | Model 2 | Model 3 | Model 1 | Model 2 | Model 3 | Model 1 | Model 2 | Model 3 |
| *Within* |  |  |  |  |  |  |  |  |  |
| Membership *_t-1_* | 0.068*  [0.001, 0.136] | 0.035  [-0.036, 0.104] | 0.059  [-0.008, 0.128] | -0.044  [-0.163, 0.068] | -0.046  [-0.159, 0.070] | -0.057  [-0.148, 0.044] | 0.049  [-0.053, 0.165] | 0.038  [-0.072, 0.152] | 0.047  [-0.066, 0.151] |
| Active participation *_t-1_* | -0.058  [-0.124, 0.006] | -0.026  [-0.089, 0.039] | -0.060  [-0.124, 0.006] | -0.075  [-0.206, 0.054] | -0.059  [-0.179, 0.074] | -0.066  [-0.180, 0.048] | -0.054  [-0.186, 0.070] | -0.040  [-0.161, 0.086] | -0.050  [-0.182, 0.077] |
| Age *_t_* | -0.039**  [-0.044, -0.032] | -0.049**  [-0.055, -0.042] | -0.038**  [-0.044, -0.032] | -0.113**  [-0.121, -0.106] | -0.116**  [-0.125, -0.108] | -0.109**  [-0.115, -0.102] | -0.054**  [-0.061, -0.047] | -0.057**  [-0.065, -0.050] | -0.053**  [-0.060, -0.046] |
| Unemployed *_t_* |  | -0.157  [-0.339, 0.023] |  |  | -0.335**  [-0.585, -0.093] |  |  | -0.299**  [-0.495, -0.091] |  |
| Out of the labor market *_t_* |  | -0.226**  [-0.308, -0.144] |  |  | -0.219**  [-0.339, -0.101] |  |  | -0.154**  [-0.252, -0.051] |  |
| Income (logged) *_t_* |  | 0.071**  [0.030, 0.111] |  |  | -0.009  [-0.067, 0.049] |  |  | 0.046  [-0.002, 0.096] |  |
| Cohabiting with partner *_t_* |  | -0.217**  [-0.320, -0.110] |  |  | -0.492**  [-0.630, -0.328] |  |  | -0.173**  [-0.284, -0.057] |  |
| Children in the household *_t_* |  | -0.275**  [-0.345, -0.202] |  |  | -0.283**  [-0.418, -0.161] |  |  | -0.148*  [-0.262, -0.032] |  |
| Subjective health^a^ *_t-1_* |  |  | 0.008*  [0.001, 0.014] |  |  | 0.015**  [0.005, 0.024] |  |  | 0.008  [-0.002, 0.017] |
| Emotional well-being  *_t-1_* |  |  | 0.063*  [0.010, 0.117] |  |  | -0.028  [-0.111, 0.061] |  |  | -0.005  [-0.081, 0.070] |
| R² | 0.039 | 0.076 | 0.040 | 0.253 | 0.289 | 0.244 | 0.071 | 0.090 | 0.072 |
| *Between* |  |  |  |  |  |  |  |  |  |
| Ever member | 0.213*  [0.034, 0.398] | -0.022  [-0.195, 0.169] | 0.133  [-0.027, 0.299] | -1.990**  [-2.547, -1.462] | -1.062**  [-1.547, -0.588] | -1.356**  [-1.803, -0.910] | -0.224  [-0.492, 0.024] | -0.169  [-0.458, 0.120] | -0.174  [-0.486, 0.120] |
| Ever active | -0.416**  [-0.583, -0.254] | -0.270**  [-0.432, -0.109] | -0.390**  [-0.552, -0.229] | -1.741**  [-2.246, -1.231] | -1.060**  [-1.526, -0.596] | -1.531**  [-1.986, -1.057] | -0.481**  [-0.749, -0.215] | -0.416**  [-0.697, -0.125] | -0.518**  [-0.799, -0.234] |
| Scotland extension sample |  | -0.636**  [-0.816, -0.450] |  |  | 0.294  [-0.267. 0.888] |  |  | 0.142  [-0.210, 0.489] |  |
| Wales extension sample |  | -0.195*  [-0.380, -0.006] |  |  | -0.704**  [-1-274, -0.190] |  |  | -0.311  [-0.675, 0.053] |  |
| Northern Ireland extension sample |  | -1.169**  [-1.349, -0.983] |  |  | -1.097**  [-1.590, -0.599] |  |  | 0.116  [-0.242, 0.473] |  |
| Woman |  | -0.976**  [-1.100, -0.846] |  |  | -0.244  [-0.613, 0.117] |  |  | -0.556**  [-0.791, -0.328] |  |
| Average highest academic qualification |  | 0.001  [-0.044, 0.046] |  |  | -0.363**  [-0.499, -0.226] |  |  | -0.127**  [-0.223, -0.035] |  |
| Average occupational status |  | -0.063  [-0.132, 0.007] |  |  | -0.373**  [-0.579, -0.168] |  |  | -0.019  [-0.149, 0.112] |  |
| Average income |  | 0.389**  [0.260, 0.515] |  |  | -1.384**  [-1.725, -1.013] |  |  | -0.125  [-0.325, 0.081] |  |
| Average subjective health |  |  | 0.042**  [0.024, 0.061] |  |  | -0.198**  [-0.243, -0.153] |  |  | -0.045**  [-0.075, -0.014] |
| Average emotional well-being |  |  | 0.493**  [0.289, 0.697] |  |  | -0.505  [-1.035, 0.013] |  |  | 0.092  [-0.239, 0.415] |
| Threshold 1 | -1.610**  [-1.748, -1.464] | -2.803**  [-2.952, -2.537] | -1.657**  [-1.749, -1.546] | 1.232**  [0.851, 1.627] | 1.053**  [0.617, 1.543] | 1.364**  [1.037, 1.711] | -2.352**  [-2.520, -2.197] | -2.809**  [-3.087, -2.566] | -2.338**  [-2.587, -2.160] |
| Threshold 2 | -1.090**  [-1.224, -0.943] | -2.277**  [-2.427, -2.013] | -1.133**  [-1.226, -1.024] | N/A | N/A | N/A | .0.227**  [-0.385, -0.089] | -0.670**  [-0.956, -0.430] | -0.209**  [-0.447, -0.047] |
| Threshold 3 | 0.088  [-0.050, 0.228] | -1.098**  [-1.241, -0.829] | 0.043  [-0.047, 0.153] | N/A | N/A | N/A | N/A | N/A | N/A |
| Threshold 4 | 1.199**  [1.053, 1.339] | 0.020  [-0.125, 0.287] | 1.153**  [1.056, 1.271] | N/A | N/A | N/A | N/A | N/A | N/A |
| Residual variance (σ^2^*_u_*) | 3.215**  [2.996, 3.450} | 2.777**  [2.586, 2.983] | 3.106**  [2.900, 3.341] | 30.189**  [26.720, 33.326] | 21.808**  [19.263, 25.550] | 22.334**  [18.872, 26.055] | 3.912**  [3.485, 4.401] | 3.954**  [3.498, 4.494] | 3.941**  [3.507, 4.418] |
| R² | 0.009 | 0.153 | 0.042 | 0.077 | 0.164 | 0.114 | 0.026 | 0.064 | 0.037 |

*Note.* Cells represent unstandardized probit regression coefficients with Bayesian credibility intervals in square brackets.

^a^ Rescaled into quantile ranks.

* *p* < .05. ** *p* < .01.

*Effects of Membership and Active Participation in Voluntary Organizations on Pub Attendance and Smoking in Older Adults: Full Results*

| Predictors | Pub attendance *_t_* | | | Smoker *_t_* | | | Smoking intensity *_t_* | | |
| --- | --- | --- | --- | --- | --- | --- | --- | --- | --- |
|  | Model 1 | Model 2 | Model 3 | Model 1 | Model 2 | Model 3 | Model 1 | Model 2 | Model 3 |
| *Within* |  |  |  |  |  |  |  |  |  |
| Membership *_t-1_* | -0.054  [-0.170, 0.069] | -0.066  [-0.188, 0.054] | -0.052  [-0.177, 0.068] | -0.051  [-0.328, 0.230] | -0.032  [-0.285, 0.230] | -0.043  [-0.301, 0.209] | -0.098  [-0.385, 0.184] | -0.086  [-0.369, 0.207] | -0.102  [-0.385, 0.183] |
| Active participation *_t-1_* | 0.052  [-0.066, 0.166] | 0.082  [-0.039, 0.202] | 0.045  [-0.071, 0.155] | -0.179  [-0.494, 0.065] | -0.154  [-0.398, 0.100] | -0.166  [-0.461, 0.140] | -0.133  [-0.439, 0.179] | -0.111  [-0.450, 0.220] | -0.120  [-0.427, 0.197] |
| Age *_t_* | -0.073**  [-0.083, -0.063] | -0.070**  [-0.081, -0.058] | -0.072**  [-0.082, -0.062] | -0.143**  [-0.160, -0.125] | -0.146**  [-0.166, -0.128] | -0.140**  [-0.159, -0.123] | -0.062**  [-0.081, -0.044] | -0.057**  [-0.080, -0.034] | -0.057**  [-0.077, -0.038] |
| Employed *_t_* |  | 0.293**  [0.090, 0.489] |  |  | -0.050  [-0.479, 0.347] |  |  | 0.176  [-0.253, 0.635] |  |
| Income (logged) *_t_* |  | -0.116*  [-0.210, -0.019] |  |  | 0.002  [-0.137, 0.131] |  |  | -0.094  [-0.263, 0.069] |  |
| Cohabiting with partner *_t_* |  | -0.137*  [-0.282, -0.006] |  |  | -0.023  [-0.295, 0.232] |  |  | -0.039  [-0.375, 0.298] |  |
| Children in the household *_t_* |  | 0.068  [-0.361, 0.490] |  |  | -0.672  [-1.452, 0.087] |  |  | 0.210  [-0.651, 1.076] |  |
| Subjective health^a^ *_t-1_* |  |  | 0.004  [-0.008, 0.016] |  |  | 0.012  [-0.008, 0.034] |  |  | 0.021  [0.000, 0.042] |
| Emotional well-being  *_t-1_* |  |  | -0.025  [-0.145, 0.092] |  |  | 0.147  [-0.061, 0.375] |  |  | 0.076  [-0.166, 0.302] |
| R² | 0.100 | 0.118 | 0.100 | 0.276 | 0.286 | 0.281 | 0.072 | 0.085 | 0.079 |
| *Between* |  |  |  |  |  |  |  |  |  |
| Ever member | 0.502**  [0.180, 0.812] | 0.269  [-0.044, 0.578] | 0.540**  [0.243, 0.831] | -1.260**  [-2.177, -0.430] | -1.209*  [-2.206, -0.268] | -1.164*  [-2.049, -0.269] | -0.029  [-0.616, 0.540] | -0.230  [-1.223, 0.726] | -0.014  [-0.604, 0.570] |
| Ever active | -0.719**  [-1.036, -0.412] | -0.224  [-0.534, 0.093] | -0.733**  [-1.047, -0.433] | -2.012**  [-2.870, -1.198] | -1.920**  [-2.778, -1.007] | -1.918**  [-2.766, -1.027] | -0.908**  [-1.509, -0.317] | -1.390**  [-2.483, -0.467] | -0.906**  [-1.517, -0.301] |
| Scotland extension sample |  | -0.516**  [-0.844, -0.197] |  |  | -0.218  [-1.638, 1.013] |  |  | -0.044  [-2.305, 1.883] |  |
| Wales extension sample |  | -0.138  [-0.435, 0.168] |  |  | -2.564**  [-3.714, -1.442] |  |  | -0.705  [-2.232, 0.968] |  |
| Northern Ireland extension sample |  | -1.162**  [-1.507, -0.809] |  |  | -1.655**  [-2.794, -0.439] |  |  | 0.292  [-1.063, 1.827] |  |
| Woman |  | -1.472**  [-1.689, -1.265] |  |  | -0.227  [-0.961, 0.482] |  |  | -0.306  [-0.993, 0.505] |  |
| Average highest academic qualification |  | -0.018  [-0.112, 0.076] |  |  | -0.379  [-0.732, 0.015] |  |  | -0.257  [-0.600, 0.054] |  |
| Average occupational status |  | -0.205*  [-0.392, -0.012] |  |  | -0.042  [-1.017, 0.795] |  |  | 0.989**  [0.425, 1.489] |  |
| Average income |  | 0.359**  [0.117, 0.613] |  |  | -0.553  [-1.261, 0.151] |  |  | -0.518  [1.323, 0.265] |  |
| Average subjective health |  |  | 0.045**  [0.015, 0.075] |  |  | -0.193**  [-0.283, -0.098] |  |  | 0.003  [-0.068, 0.074] |
| Average emotional well-being |  |  | 0.372**  [0.035, 0.705] |  |  | 0.213  [-0.738, 1.131] |  |  | -0.250  [-0.892, 0.419] |
| Threshold 1 | 0.443**  [0.247, 0.616] | -0.451**  [-0.772, -0.243] | 0.498**  [0.386, 0.600] | 3.281**  [2.541, 3.766] | 2.950**  [1.988, 3.799] | 3.530**  [2.948, 4.099] | -1.270**  [-1.588, -0.967] | -1.955**  [-2.759, -1.296] | -1.277  [-1.603, -0.821] |
| Threshold 2 | 0.780**  [0.587, 0.959] | -0.111  [-0.432, 0.098] | 0.836**  [0.724, 0.936] | N/A | N/A | N/A | 1.359**  [1.062, 1.678] | 0.683  [-0.064, 1.334] | 1.375  [1.024, 1.806] |
| Threshold 3 | 1.433**  [1.229, 1.615] | 0.545**  [0.224, 0.754] | 1.487**  [1.372, 1.586] | N/A | N/A | N/A | N/A | N/A | N/A |
| Threshold 4 | 1.988**  [1.776, 2.173] | 1.103**  [0.785, 1.314] | 2.040**  [1.924, 2.144] | N/A | N/A | N/A | N/A | N/A | N/A |
| Residual variance (σ^2^*_u_*) | 3.663**  [3.214, 4.195] | 2.990**  [2.614, 3.416] | 3.575**  [3.185, 4.051] | 25.722**  [18.886, 29.983] | 26.609**  [21.478, 33.558] | 26.163**  [22.252, 31.204] | 4.545**  [3.574, 5.833] | 3.320**  [1.911, 4.898] | 4.567**  [3.663, 5.690] |
| R² | 0.016 | 0.222 | 0.040 | 0.085 | 0.141 | 0.103 | 0.045 | 0.318 | 0.053 |

*Note.* Cells represent unstandardized probit regression coefficients with Bayesian credibility intervals in square brackets.

^a^ Rescaled into quantile ranks.

* *p* < .05. ** *p* < .01.

*Effects of Attendance at Meetings and Volunteering on Pub Attendance and Smoking in Younger Adults: Full Results*

| Predictors | Pub attendance *_t_* | | | | Smoker *_t_* | | | | Smoking intensity *_t_* | | | |
| --- | --- | --- | --- | --- | --- | --- | --- | --- | --- | --- | --- | --- |
|  | Model 1 | Model 2 | Model 3 | Model 4 | Model 1 | Model 2 | Model 3 | Model 4 | Model 1 | Model 2 | Model 3 | Model 4 |
| *Within* |  |  |  |  |  |  |  |  |  |  |  |  |
| Attendance at meetings *_t_* | -0.021  [-0.056, 0.011] | -0.001  [-0.035, 0.031] | -0.023  [-0.055, 0.009] | -0.039*  [-0.072, -0.003] | -0.051  [-0.113, 0.022] | -0.053  [-0.119, 0.008] | -0.049  [-0.120, 0.022] | -0.043  [-0.111, 0.026] | -0.020  [-0.094, 0.051] | -0.024  [-0.100, 0.055] | -0.026  [-0.100, 0.047] | -0.028  [-0.102, 0.040] |
| Volunteering *_t_* | 0.026  [-0.006, 0.058] | 0.009  [-0.024, 0.040] | 0.025  [-0.007, 0.058] | 0.005  [-0.026, 0.039] | 0.003  [-0.050, 0.056] | 0.007  [-0.053, 0.062] | 0.002  [-0.052, 0.057] | 0.005  [-0.054, 0.062] | -0.019  [-0.081, 0.040] | -0.017  [-0.077, 0.041] | -0.014  [-0.072, 0.046] | -0.009  [-0.068, 0.053] |
| Age *_t_* | -0.066**  [-0.071, -0.061] | -0.042**  [-0.048, -0.037] | -0.066**  [-0.071, -0.060] | -0.064**  [-0.069, -0.059] | -0.053**  [-0.057, -0.049] | -0.048**  [-0.053, -0.044] | -0.053**  [-0.057, -0.049] | -0.051**  [-0.055, -0.047] | -0.003  [-0.007, 0.002] | -0.003  [-0.008, 0.002] | -0.003  [-0.007, 0.001] | 0.000  [-0.005, 0.005] |
| Unemployed *_t_* |  | -0.203**  [-0.316, -0.094] |  |  |  | -0.083  [-0.198, 0.033] |  |  |  | -0.171**  [-0.267, -0.077] |  |  |
| Out of the labor market *_t_* |  | -0.413**  [-0.474, -0.353] |  |  |  | -0.080*  [-0.151, -0.007] |  |  |  | 0.004  [-0.063, 0.075] |  |  |
| Income (logged) *_t_* |  | -0.002  [-0.031, 0.029] |  |  |  | -0.021  [-0.056, 0.013] |  |  |  | -0.021  [-0.058, 0.014] |  |  |
| Cohabiting with partner *_t_* |  | -0.708**  [-0.757, -0.656] |  |  |  | -0.112**  [-0.177, -0.049] |  |  |  | -0.060*  [-0.116, -0.006] |  |  |
| Children in the household *_t_* |  | -0.809**  [-0.858, -0.763] |  |  |  | -0.389**  [-0.448, -0.327] |  |  |  | -0.050  [-0.107, 0.007] |  |  |
| Subjective health^a^ *_t_* |  |  | 0.006**  [0.002, 0.011] |  |  |  | -0.015**  [-0.020, -0.009] |  |  |  | -0.005  [-0.010, 0.000] |  |
| Emotional well-being *_t_* |  |  | 0.038  [-0.001, 0.078] |  |  |  | -0.091**  [-0.137, -0.044] |  |  |  | -0.025  [-0.068, 0.019] |  |
| Church attendance *_t_* |  |  |  | -0.047*  [-0.089, -0.006] |  |  |  | -0.137**  [-0.177, -0.090] |  |  |  | -0.084**  [-0.130,-0.038] |
| Going out *_t_* |  |  |  | 0.769**  [0.726, 0.813] |  |  |  | 0.058  [-0.021, 0.132] |  |  |  | 0.081*  [0.003, 0.156] |
| Socializing *_t_* |  |  |  | 0.087**  [0.056, 0.119] |  |  |  | 0.082**  [0.036, 0.130] |  |  |  | 0.071**  [0.027, 0.116] |
| Doing sports *_t_* |  |  |  | 0.009  [-0.011, 0.028] |  |  |  | -0.077**  [-0.121, -0.042] |  |  |  | -0.031  [-0.065, 0.003] |
| Manual work *_t_* |  |  |  | -0.039**  [-0.062, -0.016] |  |  |  | -0.019  [-0.066, 0.024] |  |  |  | -0.064**  [-0.108,-0.022] |
| R² | 0.082 | 0.331 | 0.082 | 0.152 | 0.061 | 0.106 | 0.066 | 0.075 | 0.001 | 0.005 | 0.002 | 0.010 |
| *Between* |  |  |  |  |  |  |  |  |  |  |  |  |
| Average attendance | -0.109**  [-0.172, -0.048] | -0.059*  [-0.115, -0.002] | -0.118**  [-0.182, -0.056] | -0.111**  [-0.178, -0.045] | -0.820**  [-1.004, -0.651] | -0.579**  [-0.740, -0.425] | -0.779**  [-0.947, -0.610] | -0.826**  [-0.996, -0.650] | -0.122  [-0.250, 0.005] | -0.046  [-0.167, 0.078] | -0.104  [-0.228, 0.020] | -0.118  [-0.247, 0.007] |
| Average volunteering | 0.002  [-0.061, 0.066] | -0.060*  [-0.116, -0.005] | 0.004  [-0.060, 0.067] | 0.000  [-0.067, 0.068] | -0.279**  [-0.453, -0.109] | -0.159  [-0.316, 0.002] | -0.298**  [-0.473, -0.129] | -0.280**  [-0.447, -0.115] | -0.074  [-0.196, 0.057] | 0.034  [-0.084, 0.153] | -0.088  [-0.212, 0.031] | -0.073  [-0.200, 0.050] |
| Scotland ext. sample |  | -0.483**  [-0.588, -0.376] |  |  |  | 0.169  [-0.093, .435] |  |  |  | 0.457**  [0.271, 0.642] |  |  |
| Wales ext. sample |  | -0.212**  [-0.317, -0.102] |  |  |  | -0.030  [-0.313, 0.258] |  |  |  | 0.100  [-0.099, 0.294] |  |  |
| Northern Ireland ext. sample |  | -0.728**  [-0.835, -0.621] |  |  |  | -0.064  [-0.315, 0.197] |  |  |  | 0.442**  [0.253, 0.626] |  |  |
| Woman |  | -0.308**  [-0.375, -0.235] |  |  |  | -0.350**  [-0.525, -0.181] |  |  |  | -0.456**  [-0.579, -0.342] |  |  |
| Average highest academic qualification |  | 0.113**  [0.082, 0.143] |  |  |  | -0.429**  [-0.503, -0.356] |  |  |  | -.295**  [-0.347, -0.244] |  |  |
| Average occupational status |  | 0.015  [-0.027, 0.057] |  |  |  | -0.294**  [-0.395, -0.195] |  |  |  | -0.184**  [-0.254, -0.113] |  |  |
| Average income |  | 0.081*  [0.010, 0.153] |  |  |  | -0.773**  [-0.935, -0.618] |  |  |  | -0.085  [-0.196, 0.025] |  |  |
| Average subjective health |  |  | 0.058**  [0.047, 0.069] |  |  |  | -0.186**  [-0.212, -0.162] |  |  |  | -0.087**  [-0.104, -0.071] |  |
| Average emotional well-being |  |  | 0.106  [-0.016, 0.226] |  |  |  | -0.639**  [-0.891, -0.386] |  |  |  | -0.089  [-0.251, 0.076] |  |
| Threshold 1 | -2.444**  [-2.504, -2.387] | -3.718**  [-3.795, -3.620] | -2.445**  [-2.496, -2.392] | -2.570**  [-2.619, -2.510] | 1.672**  [1.566, 1.790] | 1.218**  [1.064, 1.367] | 1.671**  [1.563, 1.770] | 1.677**  [1.575, 1.781] | -1.084**  [-1.146, -1.021] | -1.241**  [-1.353, -1.141] | -1.085**  [-1.145, -1.024] | -1.096**  [-1.149,-1.033] |
| Threshold 2 | -2.001**  [-2.057, -1.951] | -3.270**  [-3.344, -3.174] | -2.004**  [-2.049, -1.954] | -2.111**  [-2.153, -2.055] | N/A | N/A | N/A | N/A | 1.103**  [1.035, 1.168] | 0.952**  [0.850, 1.053] | 1.102**  [1.038, 1.162] | 1.105**  [1.047, 1.170] |
| Threshold 3 | -0.939**  [-0.986, -0.897] | -2.168**  [-2.237, -2.072] | -0.940**  [-0.982, -0.897] | -0.995**  [-1.032, -0.953] | N/A | N/A | N/A | N/A | N/A | N/A | N/A | N/A |
| Threshold 4 | 0.391**  [0.351, 0.430] | -0.759**  [-0.823, -0.672] | 0.389**  [0.352, 0.432] | 0.410**  [0.376, 0.450] | N/A | N/A | N/A | N/A | N/A | N/A | N/A | N/A |
| Residual variance (σ^2^*_u_*) | 1.964**  [1.849, 2.097] | 1.406**  [1.321, 1.496] | 1.892**  [1.770, 2.008] | 2.196**  [2.072, 2.335] | 12.821**  [11.879, 14.128] | 11.151**  [10.313, 12.149] | 12.009**  [11.064, 12.848] | 12.937**  [12.150, 13.972] | 3.125**  [2.903, 3.356] | 2.754**  [2.555, 2.973] | 2.990**  [2.766, 3.239] | 3.190**  [2.948, 3.442] |
| R² | 0.004 | 0.090 | 0.041 | 0.004 | 0.057 | 0.155 | 0.120 | 0.057 | 0.005 | 0.147 | 0.052 | 0.005 |

*Note.* Cells represent unstandardized probit regression coefficients with Bayesian credibility intervals in square brackets.

^a^ Rescaled into quantile ranks.

* *p* < .05. ** *p* < .01.

*Effects of Attendance at Meetings and Volunteering on Pub Attendance and Smoking in Middle-Aged Adults: Full Results*

| Predictors | Pub attendance *_t_* | | | | Smoker *_t_* | | | | Smoking intensity *_t_* | | | |
| --- | --- | --- | --- | --- | --- | --- | --- | --- | --- | --- | --- | --- |
|  | Model 1 | Model 2 | Model 3 | Model 4 | Model 1 | Model 2 | Model 3 | Model 4 | Model 1 | Model 2 | Model 3 | Model 4 |
| *Within* |  |  |  |  |  |  |  |  |  |  |  |  |
| Attendance at meetings *_t_* | 0.063**  [0.024, 0.101] | 0.068**  [0.029, 0.105] | 0.064**  [0.027, 0.101] | 0.044*  [0.005, 0.083] | -0.033  [-0.144, 0.079] | -0.041  [-0.138, 0.074] | -0.046  [-0.164, 0.077] | -0.032  [-0.151, 0.092] | -0.055  [-0.169, 0.055] | -0.059  [-0.166, 0.049] | -0.061  [-0.168, 0.048] | -0.050  [-0.166, 0.064] |
| Volunteering *_t_* | -0.034  [-0.069, 0.002] | -0.025  [-0.061, 0.012] | -0.035  [-0.072, 0.003] | -0.044*  [-0.078, -0.009] | 0.008  [-0.082, 0.100] | 0.024  [-0.067, 0.107] | 0.002  [-0.090, 0.096] | 0.009  [-0.076, 0.093] | -0.058  [-0.158, 0.043] | -0.047  [-0.136, 0.048] | -0.051  [-0.143, 0.038] | -0.059  [-0.154, 0.035] |
| Age *_t_* | -0.039**  [-0.044, -0.033] | -0.049**  [-0.055, -0.042] | -0.037**  [-0.043, -0.032] | -0.041**  [-0.047, -0.035] | -0.113**  [-0.121, -0.106] | -0.118**  [-0.126, -0.110] | -0.111**  [-0.118, -0.104] | -0.111**  [-0.118, -0.104] | -0.054**  [-0.061, -0.048] | -0.058**  [-0.066, -0.051] | -0.052**  [-0.059, -0.046] | -0.055**  [-0.062,-0.049] |
| Unemployed *_t_* |  | -0.168  [-0.341, 0.023] |  |  |  | -0.323**  [-0.562, -0.076] |  |  |  | -0.270*  [-0.481, -0.047] |  |  |
| Out of the labor market *_t_* |  | -0.223**  [-0.304, -0.147] |  |  |  | -0.203**  [-0.323, -0.093] |  |  |  | -0.146*  [-0.249, -0.030] |  |  |
| Income (logged) *_t_* |  | 0.071**  [0.030, 0.112] |  |  |  | -0.010  [-0.068, 0.050] |  |  |  | 0.044  [-0.006, 0.094] |  |  |
| Cohabiting with partner *_t_* |  | -0.195**  [-0.296, -0.094] |  |  |  | -0.501**  [-0.638, -0.369] |  |  |  | -0.164**  [-0.304, -0.048] |  |  |
| Children in the household *_t_* |  | -0.280**  [-0.349, -0.207] |  |  |  | -0.305**  [-0.436, -0.167] |  |  |  | -0.150**  [-0.259, -0.036] |  |  |
| Subjective health^a^ *_t_* |  |  | 0.011**  [0.005, 0.017] |  |  |  | 0.027**  [0.018, 0.037] |  |  |  | 0.017**  [0.008, 0.026] |  |
| Emotional well-being *_t_* |  |  | 0.055*  [0.003, 0.108] |  |  |  | -0.041  [-0.125, 0.043] |  |  |  | -0.008  [-0.083, 0.067] |  |
| Church attendance *_t_* |  |  |  | 0.019  [-0.029, 0.069] |  |  |  | -0.028  [-0.109, 0.051] |  |  |  | -0.098**  [-0.171 -0.029] |
| Going out *_t_* |  |  |  | 0.555**  [0.497, 0.617] |  |  |  | -0.034  [-0.203, 0.131] |  |  |  | 0.043  [-0.099, 0.188] |
| Socializing *_t_* |  |  |  | 0.049**  [0.016, 0.084] |  |  |  | -0.006  [-0.072, 0.056] |  |  |  | 0.027  [-0.035, 0.089] |
| Doing sports *_t_* |  |  |  | 0.039**  [0.018, 0.058] |  |  |  | -0.044  [-0.095, 0.003] |  |  |  | -0.027  [-0.075, 0.023] |
| Manual work *_t_* |  |  |  | 0.068**  [0.036, 0.099] |  |  |  | 0.012  [-0.070, 0.087] |  |  |  | -0.011  [-0.081, 0.054] |
| R² | 0.039 | 0.075 | 0.041 | 0.076 | 0.254 | 0.292 | 0.260 | 0.251 | 0.073 | 0.092 | 0.076 | 0.080 |
| *Between* |  |  |  |  |  |  |  |  |  |  |  |  |
| Average attendance | -0.092*  [-0.184, -0.003] | -0.066  [-0.155, 0.022] | -0.103*  [-0.196, -0.013] | -0.096  [-0.192, 0.001] | -1.036**  [-1.407, -0.675] | -0.573**  [-0.871, -0.284] | -0.992**  [-1.360, -0.627] | -0.902**  [-1.230, -0.597] | -0.431**  [-0.635, -0.220] | -0.395**  [-0.606, -0.183] | -0.449**  [-0.659, -0.242] | -0.436**  [-0.648,-0.234] |
| Average volunteering | -0.082  [-0.168, 0.006] | -0.096*  [-0.179, -0.011] | -0.105*  [-0.193, -0.018] | -0.084  [-0.172, 0.010] | -0.984**  [-1.345, -0.640] | -0.678**  [-1.002, -0.377] | -0.864**  [-1.249, -0.511] | -0.922**  [-1.235, -0.613] | 0.011  [-0.211, 0.224] | 0.054  [-0.168, 0.281] | 0.047  [-0.174, 0.266] | 0.022  [-0.197, 0.239] |
| Scotland ext. sample |  | -0.598**  [-0.781, -0.417] |  |  |  | 0.524  [-0.040, 1.133] |  |  |  | 0.235  [-0.107, 0.579] |  |  |
| Wales ext. sample |  | -0.137  [-0.323, 0.045] |  |  |  | -0.520  [-1.069, 0.024] |  |  |  | -0.194  [-0.547, 0.158] |  |  |
| Northern Ireland ext. sample |  | -1.152**  [-1.332, -0.974] |  |  |  | -1.104**  [-1.659, -0.550] |  |  |  | 0.192  [-0.154, 0.542] |  |  |
| Woman |  | -0.975**  [-1.116, -0.848] |  |  |  | -0.224  [-0.607, 0.199] |  |  |  | -0.535**  [-0.752, -0.332] |  |  |
| Average highest academic qualification |  | 0.002  [-0.042, 0.047] |  |  |  | -0.414**  [-0.566, -0.262] |  |  |  | -0.133**  [-0.226, -0.044] |  |  |
| Average occupational status |  | -0.055  [-0.125, 0.015] |  |  |  | -0.280*  [-0.502, -0.052] |  |  |  | 0.012  [-0.119, 0.139] |  |  |
| Average income |  | 0.381**  [0.254, 0.507] |  |  |  | -1.600**  [-1.924, -1.264] |  |  |  | -0.167  [-0.371, 0.037] |  |  |
| Average subjective health |  |  | 0.046**  [0.029, 0.063] |  |  |  | -0.234**  [-0.295, -0.177] |  |  |  | -0.041**  [-0.073, -0.011] |  |
| Average emotional well-being |  |  | 0.494**  [0.290, 0.701] |  |  |  | -0.637*  [-1.266, -0.013] |  |  |  | -0.048  [-0.357, 0.280] |  |
| Threshold 1 | -1.505**  [-1.581, -1.429] | -2.572**  [-2.747, -2.397] | -1.523**  [-1.582, -1.436] | -1.554**  [-1.626, -1.499] | 3.903**  [3.636, 4.235] | 2.742**  [2.371, 3.175] | 3.938**  [3.646, 4.311] | 3.579**  [3.335, 3.856] | -2.008**  [-2.146, -1.882] | -2.462**  [-2.627, -2.242] | -1.965**  [-2.072, -1.844] | -2.014**  [-2.132, -1.909] |
| Threshold 2 | -0.983**  [-1.064, -0.910] | -2.047**  [-2.224, -1.875] | -1.000**  [-1.060, -0.914] | -1.024**  [-1.091, -0.964] | N/A | N/A | N/A | N/A | 0.123*  [0.001, 0.244] | -0.327**  [-0.489, -0.107] | 0.165**  [0.067, 0.280] | 0.121*  [0.014, 0.220] |
| Threshold 3 | 0.191**  [0.113, 0.258] | -0.863**  [-1.037, -0.704] | 0.180**  [0.120, 0.261] | 0.183**  [0.110, 0.239] | N/A | N/A | N/A | N/A | N/A | N/A | N/A | N/A |
| Threshold 4 | 1.302**  [1.220, 1.369] | 0.257**  [0.090, 0.404] | 1.292**  [1.230, 1.381] | 1.319**  [1.246, 1.381] | N/A | N/A | N/A | N/A | N/A | N/A | N/A | N/A |
| Residual variance (σ^2^*_u_*) | 3.204**  [2.986, 3.436] | 2.764**  [2.567, 2.970] | 3.117  [2.912, 3.348] | 3.364**  [3.147, 3.609] | 31.301**  [27.838, 35.865] | 24.055**  [21.159, 26.840] | 30.616**  [26.912, 35.818] | 26.418**  [23.308, 29.865] | 3.983**  [3.504, 4.531] | 3.905**  [3.440, 4.432] | 3.892**  [3.466, 4.441] | 3.968**  [3.496, 4.475] |
| R² | 0.009 | 0.154 | 0.046 | 0.009 | 0.120 | 0.191 | 0.156 | 0.117 | 0.032 | 0.068 | 0.044 | 0.032 |

*Note.* Cells represent unstandardized probit regression coefficients with Bayesian credibility intervals in square brackets.

^a^ Rescaled into quantile ranks.

* *p* < .05. ** *p* < .01.

*Effects of Attendance at Meetings and Volunteering on Pub Attendance and Smoking in Older Adults: Full Results*

| Predictors | Pub attendance *_t_* | | | | Smoker *_t_* | | | | Smoking intensity *_t_* | | | |
| --- | --- | --- | --- | --- | --- | --- | --- | --- | --- | --- | --- | --- |
|  | Model 1 | Model 2 | Model 3 | Model 4 | Model 1 | Model 2 | Model 3 | Model 4 | Model 1 | Model 2 | Model 3 | Model 4 |
| *Within* |  |  |  |  |  |  |  |  |  |  |  |  |
| Attendance at meetings *_t_* | 0.026  [-0.026, 0.078] | 0.023  [-0.029, 0.076] | 0.018  [-0.032, 0.070] | 0.003  [-0.049, 0.057] | -0.033  [-0.235, 0.170] | -0.037  [-0.262, 0.146] | -0.019  [-0.211, 0.228] | -0.039  [-0.237, 0.137] | 0.045  [-0.168, 0.245] | 0.059  [-0.155, 0.270] | 0.024  [-0.180, 0.260] | 0.036  [-0.171, 0.265] |
| Volunteering *_t_* | 0.038  [-0.016, 0.093] | 0.035  [-0.020, 0.086] | 0.032  [-0.024, 0.085] | 0.019  [-0.036, 0.074] | -0.197  [-0.413, 0.028] | -0.192  [-0.398, 0.021] | -0.187  [-0.420, 0.012] | -0.176  [-0.370, 0.014] | -0.108  [-0.355, 0.152] | -0.099  [-0.354, 0.143] | -0.103  [-0.348, 0.145] | -0.137  [-0.377, 0.133] |
| Age *_t_* | -0.071**  [-0.082, -0.061] | -0.068**  [-0.079, -0.057] | -0.069**  [-0.079, -0.058] | -0.058**  [-0.069, -0.047] | -0.155**  [-0.176, -0.136] | -0.157**  [-0.181, -0.134] | -0.145**  [-0.165, -0.123] | -0.134**  [-0.154, -0.112] | -0.064**  [-0.084, -0.044] | -0.059**  [-0.085, -0.035] | -0.056**  [-0.076, -0.036] | -0.059**  [-0.084,-0.035] |
| Employed *_t_* |  | 0.289**  [0.079, 0.492] |  |  |  | -0.048  [-0.464, 0.353] |  |  |  | 0.198  [-0.260, 0.632] |  |  |
| Income (logged) *_t_* |  | -0.118*  [-0.214, -0.016] |  |  |  | 0.011  [-0.122, 0.151] |  |  |  | -0.091  [-0.263, 0.078] |  |  |
| Cohabiting with partner *_t_* |  | -0.136*  [-0.262, -0.002] |  |  |  | -0.074  [-0.385, 0.218] |  |  |  | -0.067  [-0.377, 0.266] |  |  |
| Children in the household *_t_* |  | 0.059  [-0.343, 0.481] |  |  |  | -0.733  [-1.566, 0.123] |  |  |  | 0.201  [-0.640, 1.077] |  |  |
| Subjective health^a^ *_t_* |  |  | 0.004  [-0.006, 0.014] |  |  |  | -0.019  [-0.211, 0.228] |  |  |  | 0.028**  [0.007, 0.050] |  |
| Emotional well-being *_t_* |  |  | 0.222**  [0.099, 0.345] |  |  |  | -0.187  [-0.420, 0.012] |  |  |  | 0.129  [-0.089, 0.353] |  |
| Church attendance *_t_* |  |  |  | 0.046  [-0.033, 0.125] |  |  |  | 0.069  [-0.063, 0.183] |  |  |  | 0.008  [-0.141, 0.155] |
| Going out *_t_* |  |  |  | 0.494**  [0.386, 0.601] |  |  |  | 0.001  [-0.330, 0.339] |  |  |  | 0.020  [-0.358, 0.417] |
| Socializing *_t_* |  |  |  | 0.044  [-0.020, 0.104] |  |  |  | 0.022  [-0.116, 0.163] |  |  |  | -0.038  [-0.172, 0.096] |
| Doing sports *_t_* |  |  |  | 0.035*  [0.005, 0.064] |  |  |  | 0.009  [-0.077, 0.100] |  |  |  | 0.077  [-0.018, 0.178] |
| Manual work *_t_* |  |  |  | 0.110**  [0.057, 0.160] |  |  |  | 0.174*  [0.018, 0.347] |  |  |  | -0.007  [-0.189, 0.205] |
| R² | 0.100 | 0.117 | 0.103 | 0.129 | 0.296 | 0.303 | 0.291 | 0.290 | 0.070 | 0.084 | 0.081 | 0.091 |
| *Between* |  |  |  |  |  |  |  |  |  |  |  |  |
| Average attendance | -0.063  [-0.173, 0.047] | 0.065  [-0.045, 0.180] | -0.087  [-0.196, 0.022] | -0.061  [-0.175, 0.053] | -1.189**  [-1.753, -0.653] | -0.996**  [-1.545, -0.434] | -0.902**  [-1.263, -0.449] | -0.846**  [-1.270, -0.443] | -0.194  [-0.549, 0.156] | -0.343  [-1.016, 0.210] | -0.183  [-0.529, 0.175] | -0.196  [-0.564, 0.151] |
| Average volunteering | 0.053  [-0.068, 0.170] | -0.024  [-0.141, 0.088] | 0.024  [-0.096, 0.145] | 0.053  [-0.071, 0.178] | -0.820*  [-1.463, -0.180] | -0.574  [-1.165, 0.007] | -0.665*  [-1.263, -0.115] | -0.627**  [-1.048, -0.161] | -0.130  [-0.598, 0.322] | -0.031  [-0.558, 0.502] | -0.153  [-0.643, 0.334] | -0.131  [-0.628, 0.359] |
| Scotland ext. sample |  | -0.524**  [-0.835, -0.212] |  |  |  | -0.171  [-1.686, 1.161] |  |  |  | 0.045  [-1.461, 1.624] |  |  |
| Wales ext. sample |  | -0.155  [-0.452, 0.148] |  |  |  | 2.293**  [-3.610, -1.094] |  |  |  | -0.629  [-1.915, 0.780] |  |  |
| Northern Ireland ext. sample |  | -1.175**  [-1.522, -0.830] |  |  |  | -1.862**  [-3.212, -0.549] |  |  |  | -0.058  [-1.221, 1.235] |  |  |
| Woman |  | -1.517**  [-1.751, -1.297] |  |  |  | -0.475  [-1.305, 0.350] |  |  |  | -0.504  [-1.106, 0.190] |  |  |
| Average highest academic qualification |  | -0.035  [-0.127, 0.060] |  |  |  | -0.355  [-0.758, 0.054] |  |  |  | -0.141  [-0.465, 0.151] |  |  |
| Average occupational status |  | -0.190*  [-0.371, -0.005] |  |  |  | -0.261  [-1.058, 0.658] |  |  |  | 0.665*  [0.088, 1.171] |  |  |
| Average income |  | 0.343**  [0.107, 0.592] |  |  |  | -0.553  [-1.292, 0.219] |  |  |  | -0.623  [-1.413, 0.084] |  |  |
| Average subjective health |  |  | 0.042**  [0.012, 0.074] |  |  |  | -0.181**  [-0.276, -0.083] |  |  |  | -0.003  [-0.077, 0.071] |  |
| Average emotional well-being |  |  | 0.325  [-0.021, 0.669] |  |  |  | 0.042  [-0.846, 0.924] |  |  |  | -0.322  [-0.991, 0.319] |  |
| Threshold 1 | 0.576**  [0.476, 0.660] | -0.529**  [-0.736, -0.326] | 0.568**  [0.478, 0.658] | 0.572**  [0.459, 0.679] | 6.238**  [5.602, 6.824] | 5.208**  [4.312, 5.942] | 5.403**  [4.901, 5.869] | 4.972**  [4.393, 5.412] | -0.845**  [-1.069, -0.635] | -1.323**  [-1.767, -0.789] | -0.832**  [-1.062, -0.587] | -0.840**  [-1.097,-0.658] |
| Threshold 2 | 0.915**  [0.815, 1.000] | -0.189  [-0.397, 0.010] | 0.904**  [0.812, 0.994] | 0.917**  [0.801, 1.025] | N/A | N/A | N/A | N/A | 1.792**  [1.554, 2.037] | 1.342**  [0.911, 1.877] | 1.835**  [1.565, 2.114] | 1.838**  [1.581, 2.038] |
| Threshold 3 | 1.564**  [1.467, 1.658] | 0.465**  [0.260, 0.659] | 1.559**  [1.471, 1.654] | 1.584**  [1.462, 1.701] | N/A | N/A | N/A | N/A | N/A | N/A | N/A | N/A |
| Threshold 4 | 2.115**  [2.007, 2.222] | 1.024**  [0.822, 1.225] | 2.118**  [2.025, 2.220] | 2.151**  [2.2023, 2.283] | N/A | N/A | N/A | N/A | N/A | N/A | N/A | N/A |
| Residual variance (σ^2^*_u_*) | 3.739**  [3.328, 4.197] | 3.003**  [2.646, 3.395] | 3.672**  [3.256, 4.142] | 3.882**  [3.473, 4.341] | 35.899**  [28.873, 42.523] | 31.438**  [25.275, 37.579] | 26.270**  [21.489, 31.311] | 22.466**  [17.646, 26.251] | 4.738**  [3.723, 5.971] | 4.312**  [3.048, 5.774] | 4.885**  [3.866, 6.327] | 4.933**  [3.907, 6.300] |
| R² | 0.002 | 0.217 | 0.022 | 0.002 | 0.101 | 0.148 | 0.118 | 0.089 | 0.014 | 0.166 | 0.023 | 0.014 |

*Note.* Cells represent unstandardized probit regression coefficients with Bayesian credibility intervals in square brackets.

^a^ Rescaled into quantile ranks.

* *p* < .05. ** *p* < .01.

*Effects of Membership, Active Participation, and Volunteering on Alcohol Consumption in Two-Wave Analyses in Younger Adults: Full Results*

| Predictors | Alcohol consumption past 12 months *_2013_* | | | | | | | | Maximum ethanol consumption past 7 days *_2013_* | | | | | | | | Risk of binge drinking past 7 days *_2013_* | | | | | | | |  |
| --- | --- | --- | --- | --- | --- | --- | --- | --- | --- | --- | --- | --- | --- | --- | --- | --- | --- | --- | --- | --- | --- | --- | --- | --- | --- |
|  | Model 1 | | Model 2 | | Model 3 | | Model 4 | | Model 1 | | Model 2 | | Model 3 | | Model 4 | | Model 1 | | Model 2 | | Model 3 | | Model 4 | |  |
| Lagged  outcome *_2010_* | | 0.603** (0.032) | | 0.579**  (0.033) | | 0.600**  (0.032) | | 0.603**  (0.032) | | 0.299**  (0.028) | | 0.264**  (0.029) | | 0.297**  (0.028) | | 0.293**  (0.028) | | 0.390**  (0.045) | | 0.377**  (0.046) | | 0.389**  (0.045) | | 0.384**  (0.045) | |
| Membership *_2011_* | | -0.032  (0.108) | | -0.080  (0.110) | | -0.042  (0.109) | | -0.023  (0.109) | | -0.006  (0.171) | | -0.038  (0.174) | | -0.011  (0.171) | | 0.046  (0.171) | | 0.056  (0.140) | | 0.070  (0.150) | | 0.052  (0.140) | | 0.093  (0.141) | |
| Active participation *_2011_* | | 0.018  (0.117) | | 0.066  (0.114) | | 0.011  (0.115) | | 0.030  (0.115) | | -0.328  (0.184) | | -0.215  (0.181) | | -0.318  (0.181) | | -0.256  (0.181) | | -0.397*  (0.153) | | -0.332*  (0.160) | | -0.394*  (0.152) | | -0.341*  (0.153) | |
| Volunteering *_2010_* | | -0.003  (0.139) | | -0.031  (0.045) | | -0.034  (0.044) | | -0.032  (0.044) | | 0.217  (0.189) | | -0.013  (0.065) | | 0.008  (0.066) | | 0.028  (0.066) | | 0.004  (0.164) | | -0.088  (0.056) | | -0.067  (0.055) | | -0.050  (0.055) | |
| Volunteering squared *_2010_* | | -0.013  (0.058) | | – | | – | | – | | -0.092  (0.078) | | – | | – | | – | | -0.032  (0.066) | | – | | – | | – | |
| Scotland ext. sample | |  | | -0.214  (0.111) | |  | |  | |  | | -0.282  (0.195) | |  | |  | |  | | -0.299  (0.193) | |  | |  | |
| Wales ext. sample | |  | | -0.078  (0.102) | |  | |  | |  | | -0.322*  (0.154) | |  | |  | |  | | -0.251  (0.146) | |  | |  | |
| Northern Ireland ext. sample | |  | | -0.141  (0.107) | |  | |  | |  | | -0.536**  (0.174) | |  | |  | |  | | -0.368*  (0.160) | |  | |  | |
| Woman | |  | | -0.363**  (0.034) | |  | |  | |  | | -0.453**  (0.124) | |  | |  | |  | | -0.065  (0.111) | |  | |  | |
| Highest educational attainment *_2011_* | |  | | -0.008  (0.034) | |  | |  | |  | | -0.009  (0.053) | |  | |  | |  | | -0.048  (0.047) | |  | |  | |
| Occupational status *_2011_* | |  | | 0.083*  (0.039) | |  | |  | |  | | 0.047  (0.062) | |  | |  | |  | | 0.046  (0.056) | |  | |  | |
| Income (logged) *_2011_* | |  | | 0.032  (0.050) | |  | |  | |  | | 0.032  (0.087) | |  | |  | |  | | 0.001  (0.092) | |  | |  | |
| Unemployed *_2011_* | |  | | -0.115  (0.205) | |  | |  | |  | | -0.261  (0.319) | |  | |  | |  | | -0.210  (0.299) | |  | |  | |
| Out of the labor market *_2011_* | |  | | 0.113  (0.174) | |  | |  | |  | | -0.177  (0.270) | |  | |  | |  | | -0.246  (0.251) | |  | |  | |
| Cohabiting with partner *_2011_* | |  | | -0.165  (0.084) | |  | |  | |  | | -0.327*  (0.139) | |  | |  | |  | | -0.352**  (0.125) | |  | |  | |
| Children in the household *_2011_* | |  | | 0.106  (0.086) | |  | |  | |  | | -0.006  (0.137) | |  | |  | |  | | 0.023  (0.126) | |  | |  | |
| Subjective health^a^ *_2011_* | |  | |  | | 0.023**  (0.008) | |  | |  | |  | | 0.015  (0.013) | |  | |  | |  | | 0.012  (0.012) | |  | |
| Emotional well-being *_2011_* | |  | |  | | -0.035  (0.080) | |  | |  | |  | | -0.047  (0.120) | |  | |  | |  | | -0.121  (0.110) | |  | |
| Church attendance *_2012_* | |  | |  | |  | | -0.015  (0.038) | |  | |  | |  | | -0.133*  (0.056) | |  | |  | |  | | -0.117*  (0.051) | |
| Intercept | | 1.595**  (0.150) | | 1.550**  (0.429) | | 1.424**  (0.293) | | 1.611**  (0.161) | | 1.926**  (0.131) | | 2.226**  (0.682) | | 1.791**  (0.369) | | 2.111**  (0.157) | | N/A | | N/A | | N/A | | N/A | |
| Threshold 1 | | N/A | | N/A | | N/A | | N/A | | N/A | | N/A | | N/A | | N/A | | 0.826**  (0.137) | | 0.411  (0.707) | | 0.636  (0.349) | | 0.613**  (0.154) | |
| Threshold 2 | | N/A | | N/A | | N/A | | N/A | | N/A | | N/A | | N/A | | N/A | | 1.191**  (0.140) | | 0.783  (0.708) | | 1.001**  (0.350) | | 0.980**  (0.156) | |
| Threshold 3 | | N/A | | N/A | | N/A | | N/A | | N/A | | N/A | | N/A | | N/A | | 1.795**  (0.145) | | 1.396*  (0.709) | | 1.606**  (0.353) | | 1.586**  (0.162) | |
| Residual variance (σ^2^*_u_*) | | 1.861**  (0.087) | | 1.797**  (0.084) | | 1.848**  (0.087) | | 1.853**  (0.087) | | 4.735**  (0.102) | | 4.591**  (0.106) | | 4.735**  (0.101) | | 4.714**  (0.102) | | N/A | | N/A | | N/A | | N/A | |
| R² | | 0.291 | | 0.315 | | 0.296 | | 0.294 | | 0.091 | | 0.116 | | 0.091 | | 0.095 | | 0.086 | | 0.106 | | 0.087 | | 0.091 | |

*Note.* Cells represent unstandardized linear regression coefficients with standard errors in parentheses.

^a^ Rescaled into quantile ranks.

* *p* < .05. ** *p* < .01.

*Effects of Membership, Active Participation, and Volunteering on Alcohol Consumption in Two-Wave Analyses in Middle-Aged Adults: Full Results*

| Predictors | Alcohol consumption past 12 months *_2013_* | | | | | | | | Maximum ethanol consumption past 7 days *_2013_* | | | | | | | | Risk of binge drinking past 7 days *_2013_* | | | | | | | |  |
| --- | --- | --- | --- | --- | --- | --- | --- | --- | --- | --- | --- | --- | --- | --- | --- | --- | --- | --- | --- | --- | --- | --- | --- | --- | --- |
|  | Model 1 | | Model 2 | | Model 3 | | Model 4 | | Model 1 | | Model 2 | | Model 3 | | Model 4 | | Model 1 | | Model 2 | | Model 3 | | Model 4 | |  |
| Lagged  outcome *_2010_* | | 0.834**  (0.017) | | 0.806**  (0.018) | | 0.829**  (0.017) | | 0.833**  (0.017) | | 0.585**  (0.023) | | 0.543**  (0.025) | | 0.576**  (0.024) | | 0.585**  (0.024) | | 0.923**  (0.048) | | 0.916**  (0.050) | | 0.918**  (0.049) | | 0.923**  (0.048) | |
| Membership *_2011_* | | 0.139  (0.073) | | 0.061  (0.073) | | 0.117  (0.072) | | 0.140  (0.073) | | 0.201  (0.103) | | 0.015  (0.104) | | 0.154  (0.104) | | 0.202  (0.104) | | 0.289*  (0.120) | | 0.094  (0.126) | | 0.250*  (0.122) | | 0.288*  (0.121) | |
| Active participation *_2011_* | | -0.027  (0.075) | | -0.032  (0.074) | | -0.031  (0.074) | | -0.025  (0.076) | | 0.115  (0.109) | | 0.076  (0.108) | | 0.103  (0.109) | | 0.116  (0.114) | | -0.015  (0.127) | | -0.060  (0.130) | | -0.017  (0.127) | | -0.009  (0.131) | |
| Volunteering *_2010_* | | 0.021  (0.082) | | 0.041  (0.029) | | 0.035  (0.029) | | 0.040  (0.030) | | -0.043  (0.122) | | 0.025  (0.042) | | 0.023  (0.042) | | 0.030  (0.042) | | -0.050  (0.145) | | 0.011  (0.049) | | 0.016  (0.046) | | 0.022  (0.047) | |
| Volunteering squared *_2010_* | | 0.007  (0.033) | | – | | – | | – | | 0.031  (0.049) | | – | | – | | – | | 0.029  (0.057) | | – | | – | | – | |
| Scotland ext. sample | |  | | -0.047  (0.086) | |  | |  | |  | | 0.161  (0.125) | |  | |  | |  | | 0.088  (0.144) | |  | |  | |
| Wales ext. sample | |  | | -0.041  (0.098) | |  | |  | |  | | -0.087  (0.130) | |  | |  | |  | | -0.156  (0.162) | |  | |  | |
| Northern Ireland ext. sample | |  | | -0.188*  (0.089) | |  | |  | |  | | -0.324*  (0.131) | |  | |  | |  | | -0.252  (0.153) | |  | |  | |
| Woman | |  | | -0.126*  (0.062) | |  | |  | |  | | -0.356*  (0.086) | |  | |  | |  | | 0.079  (0.101) | |  | |  | |
| Highest educational attainment *_2011_* | |  | | 0.015  (0.026) | |  | |  | |  | | 0.079*  (0.035) | |  | |  | |  | | 0.039  (0.041) | |  | |  | |
| Occupational status *_2011_* | |  | | 0.039  (0.031) | |  | |  | |  | | 0.062  (0.044) | |  | |  | |  | | 0.071  (0.051) | |  | |  | |
| Income (logged) *_2011_* | |  | | 0.046  (0.053) | |  | |  | |  | | 0.115  (0.067) | |  | |  | |  | | 0.195*  (0.088) | |  | |  | |
| Unemployed *_2011_* | |  | | -0.386  (0.353) | |  | |  | |  | | -0.347  (0.487) | |  | |  | |  | | -0.525  (0.583) | |  | |  | |
| Out of the labor market *_2011_* | |  | | -0.070  (0.154) | |  | |  | |  | | -0.023  (0.219) | |  | |  | |  | | -0.100  (0.259) | |  | |  | |
| Cohabiting with partner *_2011_* | |  | | -0.002  (0.077) | |  | |  | |  | | 0.024  (0.123) | |  | |  | |  | | 0.055  (0.141) | |  | |  | |
| Children in the household *_2011_* | |  | | 0.020  (0.066) | |  | |  | |  | | 0.149  (0.091) | |  | |  | |  | | 0.121  (0.105) | |  | |  | |
| Subjective health^a^ *_2011_* | |  | |  | | 0.018**  (0.006) | |  | |  | |  | | 0.032**  (0.008) | |  | |  | |  | | 0.020*  (0.010) | |  | |
| Emotional well-being *_2011_* | |  | |  | | -0.058  (0.066) | |  | |  | |  | | -0.029  (0.093) | |  | |  | |  | | 0.043  (0.108) | |  | |
| Church attendance *_2012_* | |  | |  | |  | | -0.010  (0.028) | |  | |  | |  | | -0.012  (0.040) | |  | |  | |  | | -0.013  (0.045) | |
| Intercept | | 0.532**  (0.093) | | 0.295  (0.391) | | 0.548**  (0.200) | | 0.567**  (0.117) | | 0.837**  (0.108) | | -0.108  (0.504) | | 0.656*  (0.262) | | 0.894**  (0.135) | | N/A | | N/A | | N/A | | N/A | |
| Threshold 1 | | N/A | | N/A | | N/A | | N/A | | N/A | | N/A | | N/A | | N/A | | 2.041**  (0.138) | | 3.813**  (0.668) | | 2.336**  (0.319) | | 1.980**  (0.162) | |
| Threshold 2 | | N/A | | N/A | | N/A | | N/A | | N/A | | N/A | | N/A | | N/A | | 2.730**  (0.149) | | 4.515**  (0.671) | | 3.026**  (0.322) | | 2.669**  (0.172) | |
| Threshold 3 | | N/A | | N/A | | N/A | | N/A | | N/A | | N/A | | N/A | | N/A | | 3.767**  (0.167) | | 5.564**  (0.672) | | 4.064**  (0.331) | | 3.706**  (0.189) | |
| Residual variance (σ^2^*_u_*) | | 1.514**  (0.084) | | 1.487**  (0.082) | | 1.506**  (0.200) | | 1.513**  (0.084) | | 3.032**  (0.107) | | 2.909**  (0.103) | | 3.005**  (0.107) | | 3.032**  (0.107) | | N/A | | N/A | | N/A | | N/A | |
| R² | | 0.611 | | 0.618 | | 0.613 | | 0.612 | | 0.309 | | 0.336 | | 0.315 | | 0.309 | | 0.284 | | 0.304 | | 0.286 | | 0.284 | |

*Note.* Cells represent unstandardized linear regression coefficients with standard errors in parentheses.

^a^ Rescaled into quantile ranks.

* *p* < .05. ** *p* < .01.

*Effects of Membership, Active Participation, and Volunteering on Alcohol Consumption in Two-Wave Analyses in Older Adults: Full Results*

| Predictors | Alcohol consumption past 12 months *_2013_* | | | | | | | | Maximum ethanol consumption past 7 days *_2013_* | | | | | | | | Risk of binge drinking past 7 days *_2013_* | | | | | | | |  |
| --- | --- | --- | --- | --- | --- | --- | --- | --- | --- | --- | --- | --- | --- | --- | --- | --- | --- | --- | --- | --- | --- | --- | --- | --- | --- |
|  | Model 1 | | Model 2 | | Model 3 | | Model 4 | | Model 1 | | Model 2 | | Model 3 | | Model 4 | | Model 1 | | Model 2 | | Model 3 | | Model 4 | |  |
| Lagged  outcome *_2010_* | | 0.821**  (0.025) | | 0.784**  (0.030) | | 0.813**  (0.025) | | 0.821**  (0.025) | | 0.633**  (0.026) | | 0.587**  (0.029) | | 0.623**  (0.027) | | 0.635**  (0.026) | | 1.304**  (0.094) | | 1.303**  (0.099) | | 1.298**  (0.096) | | 1.304**  (0.095) | |
| Membership *_2011_* | | -0.116  (0.127) | | -0.190  (0.129) | | -0.137  (0.128) | | -0.142  (0.132) | | 0.089  (0.138) | | 0.002  (0.140) | | 0.054  (0.138) | | 0.073  (0.141) | | 0.352  (0.203) | | 0.200  (0.207) | | 0.306  (0.205) | | 0.322  (0.204) | |
| Active participation *_2011_* | | 0.224  (0.131) | | 0.201  (0.132) | | 0.191  (0.131) | | 0.184  (0.134) | | -0.011  (0.137) | | 0.037  (0.133) | | -0.020  (0.134) | | -0.025  (0.142) | | 0.042  (0.200) | | 0.030  (0.214) | | 0.025  (0.202) | | 0.003  (0.208) | |
| Volunteering *_2010_* | | -0.110  (0.120) | | 0.046  (0.041) | | 0.042  (0.040) | | 0.050  (0.040) | | 0.056  (0.149) | | 0.007  (0.047) | | 0.019  (0.046) | | 0.028  (0.047) | | -0.117  (0.224) | | -0.010  (0.070) | | 0.020  (0.067) | | 0.024  (0.067) | |
| Volunteering squared *_2010_* | | 0.064  (0.044) | | – | | – | | – | | -0.010  (0.057) | | – | | – | | – | | 0.056  (0.083) | | – | | – | | – | |
| Scotland ext. sample | |  | | -0.167  (0.149) | |  | |  | |  | | -0.153  (0.165) | |  | |  | |  | | 0.090  (0.251) | |  | |  | |
| Wales ext. sample | |  | | -0.037  (0.126) | |  | |  | |  | | 0.030  (0.133) | |  | |  | |  | | 0.064  (0.208) | |  | |  | |
| Northern Ireland ext. sample | |  | | 0.008  (0.145) | |  | |  | |  | | -0.273  (0.154) | |  | |  | |  | | -0.323  (0.258) | |  | |  | |
| Woman | |  | | -0.154  (0.107) | |  | |  | |  | | -0.167  (0.114) | |  | |  | |  | | 0.116  (0.173) | |  | |  | |
| Highest educational attainment *_2011_* | |  | | 0.084*  (0.035) | |  | |  | |  | | 0.141**  (0.033) | |  | |  | |  | | 0.149  (0.052) | |  | |  | |
| Occupational status *_2011_* | |  | | -0.081  (0.104) | |  | |  | |  | | -0.157  (0.122) | |  | |  | |  | | -0.205  (0.191) | |  | |  | |
| Income (logged) *_2011_* | |  | | 0.126  (0.080) | |  | |  | |  | | 0.047  (0.074) | |  | |  | |  | | 0.262*  (0.126) | |  | |  | |
| Employed *_2011_* | |  | | 0.224  (0.431) | |  | |  | |  | | 0.705  (0.510) | |  | |  | |  | | 0.874  (0.793) | |  | |  | |
| Cohabiting with partner *_2011_* | |  | | 0.150  (0.111) | |  | |  | |  | | 0.247*  (0.112) | |  | |  | |  | | 0.230  (0.179) | |  | |  | |
| Children in the household *_2011_* | |  | | -0.054  (0.656) | |  | |  | |  | | 0.024  (0.565) | |  | |  | |  | | -0.446  (1.631) | |  | |  | |
| Subjective health^a^ *_2011_* | |  | |  | | 0.021*  (0.010) | |  | |  | |  | | 0.023*  (0.011) | |  | |  | |  | | 0.026  (0.016) | |  | |
| Emotional well-being *_2011_* | |  | |  | | -0.073  (0.133) | |  | |  | |  | | 0.147  (0.131) | |  | |  | |  | | 0.160  (0.221) | |  | |
| Church attendance *_2012_* | |  | |  | |  | | 0.020  (0.039) | |  | |  | |  | | 0.023  (0.044) | |  | |  | |  | | 0.028  (0.058) | |
| Intercept | | 0.370**  (0.116) | | -0.482  (0.535) | | 0.528  (0.396) | | 0.403**  (0.136) | | 0.445**  (0.112) | | -0.198  (0.517) | | -0.171  (0.381) | | 0.380**  (0.128) | | N/A | | N/A | | N/A | | N/A | |
| Threshold 1 | | N/A | | N/A | | N/A | | N/A | | N/A | | N/A | | N/A | | N/A | | 3.331**  (0.235) | | 5.741**  (0.922) | | 3.975**  (0.698) | | 3.326**  (0.254) | |
| Threshold 2 | | N/A | | N/A | | N/A | | N/A | | N/A | | N/A | | N/A | | N/A | | 4.482**  (0.273) | | 6.913**  (0.938) | | 5.128**  (0.719) | | 4.475**  (0.287) | |
| Threshold 3 | | N/A | | N/A | | N/A | | N/A | | N/A | | N/A | | N/A | | N/A | | 5.947**  (0.334) | | 8.399**  (0.961) | | 6.954**  (0.744) | | 5.939**  (0.339) | |
| Residual variance (σ^2^*_u_*) | | 2.159**  (0.172) | | 2.108**  (0.160) | | 2.152**  (0.172) | | 2.160**  (0.172) | | 2.433**  (0.118) | | 2.322**  (0.112) | | 2.406**  (0.118) | | 2.428**  (0.118) | | N/A | | N/A | | N/A | | N/A | |
| R² | | 0.604 | | 0.614 | | 0.606 | | 0.604 | | 0.392 | | 0.423 | | 0.397 | | 0.393 | | 0.328 | | 0.364 | | 0.335 | | 0.328 | |

*Note.* Cells represent unstandardized ordered logistic regression coefficients with standard errors in parentheses.

^a^ Rescaled into quantile ranks.

* *p* < .05. ** *p* < .01.

**Appendix B**

*Effects of the Rating Scale of Volunteering on Smoking*

| Predictors | Smoker *_t_* | | | Smoking intensity *_t_* | | |
| --- | --- | --- | --- | --- | --- | --- |
|  | Age 14–29 | Age 40–50 | Age 65–75 | Age 14–29 | Age 40–50 | Age 65–75 |
|  |  |  |  |  |  |  |
| *Within* |  |  |  |  |  |  |
| Volunteering *_t_* | -0.027  [-0.087, 0.029] | -0.012  [-0.092, 0.088] | -0.081  [-0.289, 0.112] | -0.013  [-0.076, 0.047] | -0.073  [-0.162, 0.025] | -0.031  [-0.286, 0.212] |
| Type of rating scale | -0.192**  [-0.270, -0.112] | -0.113  [-0.242, 0.016] | 0.586**  [0.170, 1.060] | -0.274**  [-0.359, -0.188] | -0.258**  [-0.396, -0.122] | 0.407  [-0.157, 0.985] |
| Volunteering *_t_* *Type of rating scale | 0.020  [-0.100, 0.140] | 0.003  [-0.191, 0.180] | -0.832**  [-1.710, -0.208] | -0.074  [-0.204, 0.056] | -0.041  [-0.261, 0.191] | -0.591  [-1.513, 0.458] |
| Age *_t_* | -0.048**  [-0.053, -0.044] | -0.108**  [-0.116, -0.100] | -0.158**  [-0.187, -0.139] | 0.004  [-0.002, 0.009] | -0.048**  [-0.056, -0.042] | -0.070**  [-0.092, -0.049] |

*Note.* Age groups were defined on the basis of participants’ age in 1992. Cells represent unstandardized probit regression coefficients with Bayesian credibility intervals in square brackets. Type of rating scale = binary indicator for the waves at which the 9-point rating scale of volunteering (vs. 5-point scale) was administered.

* *p* < .05. ** *p* < .01.

**Appendix C**

*Separate Analyses by Sex: Effects of Membership and Active Participation in Voluntary Organizations on Pub Attendance and Smoking in Men*

| Predictors | Pub attendance *_t_* | | | Smoker *_t_* | | | Smoking intensity *_t_* | | |
| --- | --- | --- | --- | --- | --- | --- | --- | --- | --- |
|  | Model 1 | Model 2 | Model 3 | Model 1 | Model 2 | Model 3 | Model 1 | Model 2 | Model 3 |
|  | Age 14–29 | | | | | | | | |
| *Within* |  |  |  |  |  |  |  |  |  |
| Membership *_t-1_* | 0.021  [-0.066, 0.113] | 0.025  [-0.058, 0.113] | 0.019  [-0.075, 0.108] | 0.039  [-0.063, 0.136] | 0.001  [-0.104, 0.103] | 0.037  [-0.066, 0.141] | 0.004  [-0.106, 0.113] | 0.010  [-0.105, 0.129] | -0.001  [-0.112, 0.130] |
| Active participation *_t-1_* | -0.081  [-0.188, 0.025] | -0.093  [-0.194, 0.007] | -0.076  [-0.175, 0.028] | -0.043  [-0.169, 0.084] | -0.027  [-0.156, 0.108] | -0.041  [-0.170, 0.096] | 0.087  [-0.064, 0.234] | 0.088  [-0.066, 0.245] | 0.086  [-0.071, 0.242] |
| Age *_t_* | -0.053**  [-0.061, -0.046] | -0.034**  [-0.042, -0.026] | -0.053**  [-0.061, -0.046] | -0.047**  [-0.053, -0.041] | -0.043**  [-0.049, -0.037] | -0.047**  [-0.053, -0.042] | -0.002  [-0.009, 0.004] | -0.004  [-0.011, 0.004] | -0.002  [-0.009, 0.004] |
| *Between* |  |  |  |  |  |  |  |  |  |
| Ever member | 0.111  [-0.036, 0.257] | -0.028  [-0.175, 0.123] | 0.071  [-0.069, 0.209] | -1.133**  [-1.475, -0.806] | -0.634**  [-0.952, -0.321] | -0.996**  [-1.301, -0.694] | -0.350**  [-0.572, -0.139] | -0.098  [-0.306, 0.114] | -0.322**  [-0.529, -0.115] |
| Ever active | -0.184*  [-0.326, -0.039] | -0.158*  [-0.295, -0.017] | -0.202**  [-0.344, -0.058] | -0.799**  [-1.143, -0.461] | -0.438**  [-0.763, -0.119] | -0.784**  [-1.118, -0.458] | -0.387**  [-0.620, -0.158] | -0.242*  [-0.460, -0.023] | -0.371**  [-0.594, -0.145] |
|  | Age 40–50 | | | | | | | | |
| *Within* |  |  |  |  |  |  |  |  |  |
| Membership *_t-1_* | -0.022  [-0.123, 0.087] | -0.046  [-0.157, 0.063] | -0.033  [-0.138, 0.065] | 0.002  [-0.153, 0.170] | -0.005  [-0.165, 0.168] | 0.012  [-0.161, 0.158] | 0.153  [-0.028, 0.340] | 0.140  [-0.052, 0.311] | 0.160  [-0.013, 0.342] |
| Active participation *_t-1_* | -0.048  [-0.145, 0.050] | -0.030  [-0.130, 0.069] | -0.056  [-0.152, 0.046] | -0.127  [-0.323, 0.055] | -0.133  [-0.327, 0.055] | -0.105  [-0.277, 0.051] | -0.152  [-0.361, 0.048] | -0.127  [-0.324, 0.071] | -0.168  [-0.365, 0.035] |
| Age *_t_* | -0.033**  [-0.042, -0.024] | -0.042**  [-0.052, -0.033] | -0.032**  [-0.040, -0.023] | -0.093**  [-0.102, -0.084] | -0.100**  [-0.112, -0.088] | -0.089**  [-0.098, -0.079] | -0.047**  [-0.057, -0.036] | -0.046**  [-0.058, -0.034] | -0.047**  [-0.058, -0.037] |
| *Between* |  |  |  |  |  |  |  |  |  |
| Ever member | 0.273*  [0.007, 0.547] | 0.055  [-0.235, 0.356] | 0.204  [-0.060, 0.463] | -1.756**  [-2.502, -1.009] | -0.958**  [-1.670, -0.233] | -1.358**  [-2.013, -0.724] | -0.321  [-0.767, 0.152] | -0.146  [-0.626, 0.338] | -0.267  [-0.716, 0.200] |
| Ever active | -0.415**  [-0.674, -0.152] | -0.446**  [-0.709, -0.181] | -0.414**  [-0.672, -0.164] | -1.125**  [-1.839, -0.434] | -0.546  [-1.180, 0.127] | -0.924**  [-1.519, -0.322] | -0.747**  [-1.177, -0.310] | -0.667**  [-1.130, -0.227] | -0.768**  [-1.212, -0.319] |
|  | Age 65–75 | | | | | | | | |
| *Within* |  |  |  |  |  |  |  |  |  |
| Membership *_t-1_* | -0.092  [-0.265, 0.076] | -0.102  [-0.284, 0.077] | -0.087  [-0.256, 0.085] | -0.051  [-0.382, 0.296] | -0.009  [-0.402, 0.387] | -0.027  [-0.410, 0.388] | -0.086  [-0.542, 0.336] | -0.124  [-0.598, 0.340] | -0.060  [-0.522, 0.413] |
| Active participation *_t-1_* | 0.056  [-0.123, 0.233] | 0.063  [-0.135, 0.242] | 0.053  [-0.132, 0.238] | -0.192  [-0.632, 0.142] | -0.214  [-0.641, 0.206] | -0.224  [-0.638, 0.203] | -0.176  [-0.699, 0.313] | -0.153  [-0.669, 0.414] | -0.218  [-0.746, 0.314] |
| Age *_t_* | -0.079**  [-0.092, -0.064] | -0.078**  [-0.094, -0.061] | -0.077**  [-0.092, -0.062] | -0.119**  [-0.141, -0.097] | -0.128**  [-0.156, -0.104] | -0.120**  [-0.145, -0.096] | -0.081**  [-0.113, -0.051] | -0.079**  [-0.114, -0.044] | -0.073**  [-0.104, -0.043] |
| *Between* |  |  |  |  |  |  |  |  |  |
| Ever member | 0.371  [-0.072, 0.828] | 0.336  [-0.094, 0.761] | 0.293  [-0.157, 0.739] | -1.005*  [-1.975, -0.089] | -1.090  [-2.393, 0.216] | -1.199*  [-2.363, -0.048] | 0.497  [-0.300, 1.284] | 0.449  [-0.484, 1.411] | 0.487  [-0.310, 1.306] |
| Ever active | -0.586**  [-1.013, -0.159] | -0.288  [-0.765, 0.193] | -0.658**  [-1.093, -0.221] | -1.535**  [-2.524, -0.502] | -1.172  [-2.724, 0.519] | -1.409*  [-2.591, -0.266] | -1.347**  [-2.213, -0.511] | -2.297**  [-3.742, -1.092] | -1.340**  [-2.212, -0.482] |

*Note.* Age groups were defined on the basis of participants’ age in 1996 for pub attendance and in 1992 for smoking. Cells represent unstandardized probit regression coefficients with Bayesian credibility intervals in square brackets. Model 1 = without control variables. Model 2 = adjusted for employment status, income, cohabiting with partner, and having underage children in the household at the within level and for sample origin, sex, average educational attainment, average occupational status, and average income at the between level. Model 3 = adjusted for subjective health and emotional well-being at both levels.

* *p* < .05. ** *p* < .01.

*Separate Analyses by Sex: Effects of Membership and Active Participation in Voluntary Organizations on Pub Attendance and Smoking in Women*

| Predictors | Pub attendance *_t_* | | | Smoker *_t_* | | | Smoking intensity *_t_* | | |
| --- | --- | --- | --- | --- | --- | --- | --- | --- | --- |
|  | Model 1 | Model 2 | Model 3 | Model 1 | Model 2 | Model 3 | Model 1 | Model 2 | Model 3 |
|  | Age 14–29 | | | | | | | | |
| *Within* |  |  |  |  |  |  |  |  |  |
| Membership *_t-1_* | -0.098*  [-0.167, -0.022] | -0.114**  [-0.189, -0.044] | -0.106**  [-0.178, -0.030] | -0.010  [-0.117, 0.096] | -0.014  [-0.119, 0.099] | -0.005  [-0.108, 0.088] | -0.024  [-0.119, 0.079] | -0.019  [-0.112, 0.075] | -0.017  [-0.113, 0.085] |
| Active participation *_t-1_* | -0.108**  [-0.179, -0.041] | -0.068  [-0.137, 0.004] | -0.109**  [-0.181, -0.038] | -0.001  [-0.105, 0.101] | 0.029  [-0.080, 0.142] | 0.008  [-0.099, 0.116] | -0.036  [-0.148, 0.072] | -0.039  [-0.155, 0.075] | -0.044  [-0.159, 0.069] |
| Age *_t_* | -0.076**  [-0.083, -0.070] | -0.049**  [-0.056, -0.042] | -0.076**  [-0.082, -0.070] | -0.059**  [-0.064, -0.054] | -0.056**  [-0.062, -0.050] | -0.060**  [-0.066, -0.055] | -0.005  [-0.010, 0.002] | -0.003  [-0.010, 0.003] | -0.005  [-0.010, 0.001] |
| *Between* |  |  |  |  |  |  |  |  |  |
| Ever member | 0.259**  [0.118, 0.394] | -0.030  [-0.155, 0.099] | 0.230**  [0.093, 0.363] | -1.296**  [-1.605, -0.981] | -0.591**  [-0.905, -0.283] | -1.160**  [-1.471, -0.843] | -0.524**  [-0.740, -0.327] | -0.168  [-0.376, 0.034] | -0.427**  [-0.629, -0.226] |
| Ever active | -0.632**  [-0.762, -0.502] | -0.304**  [-0.418, -0.186] | -0.625**  [-0.751, -0.498] | -0.788**  [-1.080, -0.494] | -0.672**  [-0.964, -0.377] | -0.772**  [-1.078, -0.477] | -0.041  [-0.255, 0.161] | 0.001  [-0.196, 0.206] | -0.010  [-0.214, 0.190] |
|  | Age 40–50 | | | | | | | | |
| *Within* |  |  |  |  |  |  |  |  |  |
| Membership *_t-1_* | 0.114**  [0.030, 0.199] | 0.081  [-0.003, 0.167] | 0.106*  [0.018, 0.191] | -0.106  [-0.263, 0.037] | -0.088  [-0.261, 0.086] | -0.098  [-0.243, 0.088] | -0.025  [-0.162, 0.118] | -0.026  [-0.177, 0.109] | -0.034  [-0.172, 0.114] |
| Active participation *_t-1_* | -0.061  [-0.143, 0.021] | -0.023  [-0.102, 0.057] | -0.067  [-0.148, 0.014] | -0.039  [-0.209, 0.148] | 0.011  [-0.167, 0.198] | -0.013  [-0.211, 0.164] | 0.017  [-0.143, 0.159] | 0.034  [-0.134, 0.196] | 0.021  [-0.132, 0.191] |
| Age *_t_* | -0.042**  [-0.050, -0.035] | -0.053**  [-0.061, -0.044] | -0.043**  [-0.050, -0.035] | -0.135**  [-0.145, -0.124] | -0.141**  [-0.154, -0.128] | -0.133**  [-0.142, -0.123] | -0.059**  [-0.067, -0.051] | -0.065**  [-0.075, -0.055] | -0.057**  [-0.066, -0.049] |
| *Between* |  |  |  |  |  |  |  |  |  |
| Ever member | 0.179  [-0.034, 0.387] | -0.032  [-0.243, 0.163] | 0.114  [-0.095, 0.334] | -2.163**  [-2.904, -1.441] | -1.529**  [-2.358, -0.688] | -1.614**  [-2.381, -0.793] | -0.217  [-0.557, 0.134] | -0.170  [-0.544, 0.195] | -0.078  [-0.449, 0.271] |
| Ever active | -0.265**  [-0.463, -0.067] | -0.133  [-0.324, 0.059] | -0.208*  [-0.403, -0.010] | -2.170**  [-2.955, -1.402] | -1.887**  [-2.688, -1.097] | -2.310**  [-3.022, -1.592] | -0.207  [-0.562, 0.148] | -0.216  [-0.573, 0.150] | -0.247  [-0.605, 0.105] |
|  | Age 65–75 | | | | | | | | |
| *Within* |  |  |  |  |  |  |  |  |  |
| Membership *_t-1_* | -0.021  [-0.186, 0.151] | -0.046  [-0.211, 0.132] | -0.023  [-0.189, 0.142] | -0.038  [-0.374, 0.308] | -0.031  [-0.416, 0.324] | -0.048  [-0.426, 0.341] | -0.106  [-0.473, 0.260] | -0.091  [-0.482, 0.295] | -0.120  [-0.509, 0.274] |
| Active participation *_t-1_* | 0.092  [-0.070, 0.245] | 0.112  [-0.047, 0.276] | 0.093  [-0.068, 0.253] | -0.204  [-0.589, 0.151] | -0.159  [-0.551, 0.213] | -0.209  [-0.611, 0.189] | -0.062  [-0.460, 0.321] | -0.089  [-0.476, 0.309] | -0.066  [-0.470, 0.338] |
| Age *_t_* | -0.067**  [-0.081, -0.052] | -0.061**  [-0.078, -0.045] | -0.069**  [-0.084, -0.054] | -0.167**  [-0.191, -0.141] | -0.172**  [-0.202, -0.143] | -0.173**  [-0.200, -0.146] | -0.052**  [-0.075, -0.026] | -0.046**  [-0.073, -0.018] | -0.048**  [-0.073, -0.023] |
| *Between* |  |  |  |  |  |  |  |  |  |
| Ever member | 0.135  [-0.303, 0.569] | 0.010  [-0.456, 0.482] | 0.109  [-0.332, 0.543] | -1.393*  [-2.686, -0.059] | -1.743  [-3.680, 0.029] | -1.638*  [-3.245, -0.055] | -0.683  [-1.509, 0.100] | -0.950  [-2.381, 0.423] | -0.575  [-1.411, 0.248] |
| Ever active | -0.048  [-0.489, 0.378] | -0.044  [-0.494, 0.398] | -0.065  [-0.488, 0.363] | -2.386**  [-3.859, -1.049] | -2.406*  [-4.251, -0.572] | -2.839**  [-4.545, -1.232] | -0.311  [-1.117, 0.521] | -0.188  [-1.611, 1.135] | -0.227  [-1.043, 0.626] |

*Note.* Age groups were defined on the basis of participants’ age in 1996 for pub attendance and in 1992 for smoking. Cells represent unstandardized probit regression coefficients with Bayesian credibility intervals in square brackets. Model 1 = without control variables. Model 2 = adjusted for employment status, income, cohabiting with partner, and having underage children in the household at the within level and for sample origin, sex, average educational attainment, average occupational status, and average income at the between level. Model 3 = adjusted for subjective health and emotional well-being at both levels.

* *p* < .05. ** *p* < .01.

*Separate Analyses by Sex: Effects of Attendance at Meetings and Volunteering on Pub Attendance and Smoking in Men*

| Predictors | Pub attendance *_t_* | | | | Smoker *_t_* | | | | Smoking intensity *_t_* | | | |
| --- | --- | --- | --- | --- | --- | --- | --- | --- | --- | --- | --- | --- |
|  | Model 1 | Model 2 | Model 3 | Model 4 | Model 1 | Model 2 | Model 3 | Model 4 | Model 1 | Model 2 | Model 3 | Model 4 |
|  | Age 14–29 | | | | | | | | | | | |
| *Within* |  |  |  |  |  |  |  |  |  |  |  |  |
| Attendance at meetings *_t_* | 0.033  [-0.023, 0.086] | 0.027  [-0.029, 0.084] | 0.030  [-0.027, 0.086] | 0.014  [-0.042, 0.070] | 0.007  [-0.104, 0.123] | 0.013  [-0.093, 0.127] | 0.011  [-0.097, 0.114] | 0.007  [-0.099, 0.118] | -0.093  [-0.218, 0.037] | -0.094  [-0.217, 0.037] | -0.098  [-0.221, 0.037] | -0.094  [-0.220, 0.027] |
| Volunteering *_t_* | 0.012  [-0.045, 0.068] | -0.003  [-0.057, 0.053] | 0.014  [-0.041, 0.067] | -0.011  [-0.067, 0.048] | -0.013  [-0.105, 0.081] | -0.024  [-0.110, 0.072] | -0.017  [-0.109, 0.073] | -0.010  [-0.103, 0.092] | -0.024  [-0.135, 0.084] | -0.029  [-0.136, 0.072] | -0.023  [-0.123, 0.085] | -0.032  [-0.132, 0.077] |
| Age *_t_* | -0.053**  [-0.061, -0.046] | -0.034**  [-0.041, -0.025] | -0.052**  [-0.060, -0.045] | -0.047**  [-0.054, -0.039] | -0.047**  [-0.052, -0.041] | -0.043**  [-0.049, -0.037] | -0.047**  [-0.053, -0.041] | -0.044**  [-0.049, -0.038] | -0.001  [-0.008, 0.006] | -0.003  [-0.010, 0.004] | -0.002  [-0.008, 0.005] | 0.004  [-0.003, 0.011] |
| *Between* |  |  |  |  |  |  |  |  |  |  |  |  |
| Average attendance | -0.020  [-0.113, 0.073] | -0.025  [-0.112, 0.063] | -0.027  [-0.122, 0.063] | -0.019  [-0.118, 0.077] | -0.823**  [-1.094, -0.590] | -0.591**  [-0.892, -0.352] | -0.738**  [-0.994, -0.488] | -0.861**  [-1.132, -0.588] | -0.148  [-0.330, 0.033] | -0.044  [-0.220, 0.129] | -0.112  [-0.290, 0.068] | -0.156  [-0.339, 0.028] |
| Average volunteering | -0.017  [-0.115, 0.085] | -0.050  [-0.138, 0.040] | -0.031  [-0.128, 0.065] | -0.015  [-0.116, 0.084] | -0.332*  [-0.578, -0.072] | -0.230  [-0.474, 0.034] | -0.329**  [-0.584, -0.080] | -0.337*  [-0.605, -0.073] | -0.135  [-0.318, 0.045] | -0.046  [-0.221, 0.139] | -0.140  [-0.322, 0.041] | -0.133  [-0.323, 0.050] |
|  | Age 40–50 | | | | | | | | | | | |
| *Within* |  |  |  |  |  |  |  |  |  |  |  |  |
| Attendance at meetings *_t_* | 0.084**  [0.024, 0.142] | 0.086**  [0.026, 0.146] | 0.084**  [0.025, 0.149] | 0.052  [-0.008, 0.113] | -0.016  [-0.179, 0.153] | -0.012  [-0.177, 0.151] | -0.013  [-0.176, 0.148] | 0.011  [-0.160, 0.171] | -0.123  [-0.299, 0.055] | -0.127  [-0.316, 0.039] | -0.134  [-0.299, 0.025] | -0.137  [-0.298, 0.058] |
| Volunteering *_t_* | -0.023  [-0.083, 0.040] | -0.015  [-0.076, 0.043] | -0.023  [-0.086, 0.036] | -0.026  [-0.086, 0.031] | 0.018  [-0.101, 0.152] | 0.031  [-0.098, 0.158] | 0.019  [-0.102, 0.142] | 0.022  [-0.097, 0.150] | -0.100  -0.246, 0.043] | -0.066  [-0.208, 0.082] | -0.097  [-0.237, 0.052] | -0.092  [-0.233, 0.060] |
| Age *_t_* | -0.032**  [-0.040, -0.023] | -0.042**  [-0.052, -0.032] | -0.031**  [-0.039, -0.022] | -0.035**  [-0.043, -0.025] | -0.092**  [-0.102, -0.083] | -0.099**  [-0.111, -0.088] | -0.090**  [-0.099, -0.080] | -0.093**  [-0.102, -0.083] | -0.050**  [-0.060, -0.039] | -0.048**  [-0.061, -0.035] | -0.047**  [-0.058, -0.036] | -0.050**  [-0.061, -0.039] |
| *Between* |  |  |  |  |  |  |  |  |  |  |  |  |
| Average attendance | 0.016  [-0.136, 0.168] | 0.018  [-0.133, 0.168] | 0.018  [-0.130, 0.164] | 0.011  [-0.149, 0.164] | -0.747**  [-1.212, -0.308] | -0.424  [-0.873, 0.009] | -0.782**  [-1.256, -0.323] | -0.739**  [-1.215, -0.294] | -0.640**  [-0.976, -0.316] | -0.595**  [-0.918, -0.268] | -0.637**  [-0.968, -0.328] | -0.656**  [-0.994, -0.319] |
| Average volunteering | -0.125  [-0.266, 0.017] | -0.132  [-0.274, 0.009] | -0.142*  [-0.286, -0.003] | -0.122  [-0.265, 0.030] | -0.955**  [-1.412, -0.487] | -0.704**  [-1.159, -0.264] | -0.903**  [-1.413, -0.453] | -0.987**  [-1.472, -0.483] | -0.011  [-0.364, 0.331] | -0.046  [-0.300, 0.397] | -0.021  [-0.372, 0.324] | -0.007  [-0.353, 0.351] |
|  | Age 65–75 | | | | | | | | | | | |
| *Within* |  |  |  |  |  |  |  |  |  |  |  |  |
| Attendance at meetings *_t_* | 0.070  [-0.014, 0.153] | 0.065  [-0.028, 0.152] | 0.063  [-0.024, 0.147] | 0.035  [-0.051, 0.120] | -0.047  [-0.330, 0.300] | -0.075  [-0.422, 0.274] | -0.056  [-0.446, 0.266] | -0.092  [-0.379, 0.248] | -0.215  [-0.627, 0.157] | -0.213  [-0.639, 0.199] | -0.270  [-0.646, 0.130] | -0.308  [-0.743, 0.118] |
| Volunteering *_t_* | -0.003  [-0.085, 0.075] | -0.004  [-0.087, 0.073] | -0.014  [-0.097, 0.068] | -0.025  [-0.106, 0.057] | -0.078  [-0.389, 0.208] | -0.099  [-0.456, 0.257] | -0.064  [-0.329, 0.248] | -0.118  [-0.413, 0.200] | -0.163  [-0.521, 0.211] | -0.145  [-0.521, 0.263] | -0.159  [-0.548, 0.225] | -0.176  [-0.614, 0.229] |
| Age *_t_* | -0.078**  [-0.093, -0.063] | -0.079**  [-0.096, -0.061] | -0.072**  [-0.088, -0.057] | -0.058**  [-0.076, -0.042] | -0.123**  [-0.148, -0.099] | -0.134**  [-0.165, -0.105] | -0.115**  [-0.141, -0.089] | -0.101**  [-0.132, -0.072] | -0.091**  [-0.126, -0.057] | -0.085**  [-0.127, -0.045] | -0.080**  [-0.114, -0.045] | -0.066**  [-0.107, -0.024] |
| *Between* |  |  |  |  |  |  |  |  |  |  |  |  |
| Average attendance | 0.099  [-0.085, 0.292] | 0.170  [-0.022, 0.374] | 0.085  [-0.105, 0.279] | 0.111  [-0.080, 0.312] | -0.632  [-1.443, 0.022] | -0.571  [-1.463, 0.255] | -0.502  [-1.081, 0.092] | -0.712*  [-1.354, -0.053] | -0.167  [-0.756, 0.392] | -0.362  [-1.209, 0.377] | -0.206  [-0.781, 0.376] | -0.183  [-0.750, 0.397] |
| Average volunteering | -0.211*  [-0.420, -0.017] | -0.230*  [-0.446, -0.014] | -0.216*  [-0.424, -0.007] | -0.214*  [-0.423, -0.005] | -1.060*  [-1.964, -0.172] | -1.098*  [-2.155, -0.085] | -0.978**  [-1.619, -0.353] | -1.089**  [-1.907, -0.237] | -0.272  [-0.996, 0.453] | -0.159  [-0.953, 0.608] | -0.273  [-0.975, 0.465] | -0.274  [-1.025, 0.505] |

*Note.* Age groups were defined on the basis of participants’ age in 1996 for pub attendance and in 1992 for smoking. Cells represent unstandardized probit regression coefficients with Bayesian credibility intervals in square brackets. Model 1 = without control variables. Model 2 = adjusted for employment status, income, cohabiting with partner, and having underage children in the household at the within level and for sample origin, sex, average educational attainment, average occupational status, and average income at the between level. Model 3 = adjusted for subjective health and emotional well-being at both levels. Model 4 = adjusted for church attendance, going out, socializing, doing sports, and manual work at the within level.

* *p* < .05. ** *p* < .01.

*Separate Analyses by Sex: Effects of Attendance at Meetings and Volunteering on Pub Attendance and Smoking in Women*

| Predictors | Pub attendance *_t_* | | | | Smoker *_t_* | | | | Smoking intensity *_t_* | | | |
| --- | --- | --- | --- | --- | --- | --- | --- | --- | --- | --- | --- | --- |
|  | Model 1 | Model 2 | Model 3 | Model 4 | Model 1 | Model 2 | Model 3 | Model 4 | Model 1 | Model 2 | Model 3 | Model 4 |
|  | Age 14–29 | | | | | | | | | | | |
| *Within* |  |  |  |  |  |  |  |  |  |  |  |  |
| Attendance at meetings *_t_* | -0.050*  [-0.089, -0.012] | -0.015  [-0.057, 0.025] | -0.051*  [-0.091, -0.010] | -0.064**  [-0.106, -0.019] | -0.092*  [-0.183, -0.006] | -0.091*  [-0.182, -0.007] | -0.088  [-0.171, 0.003] | -0.083  [-0.173, 0.006] | 0.012  [-0.086, 0.110] | 0.015  [-0.077, 0.101] | 0.012  [-0.078, 0.106] | 0.006  [-0.093, 0.102] |
| Volunteering *_t_* | 0.031  [-0.007, 0.070] | 0.014  [-0.028, 0.053] | 0.030  [-0.010, 0.069] | 0.013  [-0.028, 0.054] | 0.011  [-0.061, 0.085] | 0.010  [-0.062, 0.083] | 0.008  [-0.069, 0.073] | 0.017  [-0.070, 0.090] | -0.006  [-0.087, 0.076] | -0.018  [-0.098, 0.066] | -0.003  [-0.079, 0.074] | 0.004  [-0.081, 0.083] |
| Age *_t_* | -0.077**  [-0.083, -0.070] | -0.049**  [-0.056, -0.042] | -0.076**  [-0.083, -0.070] | -0.077**  [-0.084, -0.071] | -0.059**  [-0.065, -0.054] | -0.054**  [-0.060, -0.049] | -0.059**  [-0.065, -0.054] | -0.059**  [-0.064, -0.053] | -0.004  [-0.010, 0.001] | -0.003  [-0.009, 0.003] | -0.005  [-0.011, 0.001] | -0.003  [-0.009, 0.003] |
| *Between* |  |  |  |  |  |  |  |  |  |  |  |  |
| Average attendance | -0.154**  [-0.236, -0.071] | -0.069  [-0.142, 0.003] | -0.163**  [-0.244, -0.081] | -0.160**  [-0.246, -0.074] | -0.816**  [-1.061, -0.579] | -0.593**  [-0.817, -0.382] | -0.791**  [-1.029, -0.542] | -0.803**  [-1.026, -0.569] | -0.072  [-0.257, 0.109] | -0.065  [-0.237, 0.103] | -0.068  [-0.246, 0.104] | -0.067  [-0.252, 0.111] |
| Average volunteering | 0.033  [-0.048, 0.114] | -0.081*  [-0.152, -0.008] | 0.040  [-0.041, 0.120] | 0.038  [-0.052, 0.122] | -0.232  [-0.463, 0.010] | -0.091  [-0.310, 0.138] | -0.233*  [-0.470, -0.004] | -0.229*  [-0.446, -0.012] | -0.006  [-0.179, 0.159] | 0.115  [-0.053, 0.279] | -0.034  [-0.196, 0.132] | -0.012  [-0.181, 0.154] |
|  | Age 40–50 | | | | | | | | | | | |
| *Within* |  |  |  |  |  |  |  |  |  |  |  |  |
| Attendance at meetings *_t_* | 0.048  [-0.002, 0.097] | 0.056*  [0.008, 0.106] | 0.048  [-0.003, 0.097] | 0.036  [-0.015, 0.084] | -0.070  [-0.259. 0.104] | -0.050  [-0.200, 0.093] | -0.073  [-0.225, 0.088] | -0.059  [-0.200, 0.102] | 0.005  [-0.134, 0.140] | 0.004  [-0.132, 0.153] | -0.007  [-0.142, 0.143] | 0.005  [-0.136, 0.141] |
| Volunteering *_t_* | -0.040  [-0.085, 0.004] | -0.031  [-0.075, 0.014] | -0.039  [-0.087, 0.008] | -0.051*  [-0.097, -0.003] | -0.002  [-0.149, 0.144] | -0.005  [-0.119, 0.111] | -0.017  [-0.148, 0.141] | -0.004  [-0.137, 0.128] | -0.031  [-0.144, 0.090] | -0.024  [-0.151, 0.094] | -0.020  [-0.134, 0.095] | -0.030  [-0.141, 0.089] |
| Age *_t_* | -0.043**  [-0.050, -0.036] | -0.053**  [-0.061, -0.045] | -0.042**  [-0.050, -0.035] | -0.046**  [-0.053, .0.039] | -0.135**  [-0.145, -0.125] | -0.136**  [-0.150, -0.123] | -0.131**  [-0.142, -0.121] | -0.134**  [-0.144, -0.124] | -0.059**  [-0.067, -0.050] | -0.065**  [-0.075, -0.055] | -0.057**  [-0.066, -0.048] | -0.059**  [-0.068, -0.051] |
| *Between* |  |  |  |  |  |  |  |  |  |  |  |  |
| Average attendance | -0.131*  [-0.242, -0.022] | -0.131*  [-0.236, -0.025] | -0.163**  [-0.272, -0.057] | -0.132*  [-0.242, -0.026] | -1.182**  [-1.649, -0.738] | -0.574**  [-1.009, -0.154] | -1.002**  [-1.522, -0.536] | -1.031**  [-1.539, -0.572] | -0.239  [-0.510, 0.020] | -0.198  [-0.473, 0.077] | -0.262  [-0.535, 0.009] | -0.234  [-0.502, 0.024] |
| Average volunteering | -0.059  [-0.167, 0.045] | -0.069  [-0.173, 0.033] | -0.068  [-0.174, 0.038] | -0.062  [-0.166, 0.045] | -0.927**  [-1.360, -0.511] | -0.643**  [-1.040, -0.234] | -0.758**  [-1.205, -0.257] | -0.838**  [-1.259, -0.357] | 0.014  [-0.266, 0.293] | 0.039  [-0.251, 0.327] | 0.062  [-0.226, 0.354] | -0.003  [-0.271, 0.279] |
|  | Age 65–75 | | | | | | | | | | | |
| *Within* |  |  |  |  |  |  |  |  |  |  |  |  |
| Attendance at meetings *_t_* | -0.002  [-0.068, 0.062] | -0.002  [-0.064, 0.061] | -0.003  [-0.071, 0.061] | -0.014  [-0.086, 0.053] | -0.014  [-0.281, 0.120] | -0.051  [-0.280, 0.179] | -0.047  [-0.309, 0.207] | -0.045  [-0.253, 0.191] | 0.129  [-0.143, 0.389] | 0.164  [-0.091, 0.424] | 0.157  [-0.123, 0.425] | 0.182  [-0.104, 0.473] |
| Volunteering *_t_* | 0.066  [-0.009, 0.140] | 0.065  [-0.010, 0.140] | 0.065  [-0.008, 0.142] | 0.053  [-0.017, 0.125] | -0.217  [-0.508, 0.002] | -0.178  [-0.435, 0.050] | -0.310*  [-0.633, -0.012] | -0.286*  [-0.551, -0.042] | -0.101  [-0.450, 0.239] | -0.091  [-0.429, 0.221] | -0.093  [-0.472, 0.286] | -0.105  [-0.458, 0.238] |
| Age *_t_* | -0.065**  [-0.080, -0.050] | -0.058**  [-0.075, -0.042] | -0.065**  [-0.079, -0.049] | -0.058**  [-0.072, -0.043] | -0.170**  [-0.197, -0.145] | -0.172**  [-0.205, -0.145] | -0.185**  [-0.221, -0.154] | -0.180**  [-0.216, -0.144] | -0.051**  [-0.078, -0.026] | -0.044**  [-0.075, -0.015] | -0.044**  [-0.074, -0.016] | -0.054**  [-0.087, -0.020] |
| *Between* |  |  |  |  |  |  |  |  |  |  |  |  |
| Average attendance | -0.019  [-0.134, 0.094] | 0.002  [-0.116, 0.126] | -0.040  [-0.156, 0.078] | -0.010  [-0.123, 0.106] | -1.085**  [-1.586, -0.496] | -0.957**  [-1.502, -0.466] | -1.440**  [-2.236, -0.718] | -1.255**  [-1.901, -0.601] | -0.166  [-0.647, 0.299] | -1.416*  [-2.904, -0.228] | -0.186  [-0.682, 0.272] | -0.164  [-0.659, 0.315] |
| Average volunteering | 0.135*  [0.009, 0.265] | 0.104  [-0.029, 0.229] | 0.115  [-0.014, 0.243] | 0.135*  [0.005, 0.262] | -0.372  [-0.988, 0.181] | -0.133  [-0.656, 0.403] | -0.595  [-1.786, 0.482] | -0.734  [-1.659, 0.112] | -0.081  [-0.724, 0.573] | 0.524  [-0.402, 1.478] | -0.095  [-0.776, 0.547] | 0.067  [-0.724, 0.564] |

*Note.* Age groups were defined on the basis of participants’ age in 1996 for pub attendance and in 1992 for smoking. Cells represent unstandardized probit regression coefficients with Bayesian credibility intervals in square brackets. Model 1 = without control variables. Model 2 = adjusted for employment status, income, cohabiting with partner, and having underage children in the household at the within level and for sample origin, sex, average educational attainment, average occupational status, and average income at the between level. Model 3 = adjusted for subjective health and emotional well-being at both levels. Model 4 = adjusted for church attendance, going out, socializing, doing sports, and manual work at the within level.

* *p* < .05. ** *p* < .01.

*Separate Analyses by Sex: Effects of Membership, Active Participation, and Volunteering on Alcohol Consumption in Two-Wave Analyses in Men*

| Predictors | Alcohol consumption past 12 months *_2013_* | | | | | | | | Maximum ethanol consumption past 7 days *_2013_* | | | | | | | | Risk of binge drinking past 7 days *_2013_* | | | | | | | |  |
| --- | --- | --- | --- | --- | --- | --- | --- | --- | --- | --- | --- | --- | --- | --- | --- | --- | --- | --- | --- | --- | --- | --- | --- | --- | --- |
|  | Model 1 | | Model 2 | | Model 3 | | Model 4 | | Model 1 | | Model 2 | | Model 3 | | Model 4 | | Model 1 | | Model 2 | | Model 3 | | Model 4 | |  |
| Age 14­–29 | | | | | | | | | | | | | | | | | | | | | | | | |  |
| Lagged  outcome *_2010_* | | 0.592**  (0.043) | | 0.599**  (0.045) | | 0.595**  (0.043) | | 0.595**  (0.043) | | 0.294**  (0.045) | | 0.278**  (0.047) | | 0.297**  (0.045) | | 0.290**  (0.045) | | 0.419**  (0.066) | | 0.406**  (0.070) | | 0.427**  (0.066) | | 0.409**  (0.067) | |
| Membership *_2011_* | | -0.141  (0.158) | | -0.126  (0.162) | | -0.171  (0.157) | | -0.149  (0.158) | | -0.251  (0.260) | | -0.227  (0.268) | | -0.282  (0.257) | | -0.209  (0.260) | | 0.110  (0.202) | | 0.207  (0.218) | | 0.090  (0.204) | | 0.151  (0.204) | |
| Active participation *_2011_* | | 0.288  (0.191) | | 0.295  (0.188) | | 0.277  (0.186) | | 0.287  (0.189) | | 0.330  (0.286) | | 0.385  (0.278) | | 0.301  (0.280) | | 0.391  (0.279) | | -0.057  (0.238) | | -0.091  (0.244) | | -0.112  (0.236) | | -0.032  (0.234) | |
| Volunteering *_2010_* | | -0.027  (0.217) | | -0.031  (0.074) | | -0.025  (0.072) | | -0.038  (0.071) | | -0.079  (0.296) | | -0.041  (0.109) | | 0.004  (0.106) | | 0.010  (0.107) | | -0.236  (0.264) | | -0.069  (0.093) | | -0.042  (0.090) | | -0.035  (0.092) | |
| Volunteering² *_2010_* | | -0.003  (0.090) | | – | | – | | – | | 0.031  (0.120) | | – | | – | | – | | 0.076  (0.100) | | – | | – | | – | |
| Age 40­–50 | | | | | | | | | | | | | | | | | | | | | | | | |  |
| Lagged  outcome *_2010_* | | 0.813**  (0.027) | | 0.799**  (0.028) | | 0.809**  (0.027) | | 0.812**  (0.027) | | 0.571**  (0.036) | | 0.565**  (0.038) | | 0.563**  (0.037) | | 0.569**  (0.036) | | 0.888**  (0.074) | | 0.895**  (0.074) | | 0.889**  (0.075) | | 0.883*  (0.074) | |
| Membership *_2011_* | | 0.234  (0.120) | | 0.178  (0.123) | | 0.214  (0.119) | | 0.225  (0.120) | | 0.259  (0.148) | | 0.142  (0.152) | | 0.213  (0.149) | | 0.259  (0.148) | | 0.358  (0.183) | | 0.263  (0.200) | | 0.335  (0.187) | | 0.377*  (0.184) | |
| Active participation *_2011_* | | -0.106  (0.122) | | -0.108  (0.127) | | -0.112  (0.122) | | -0.125  (0.120) | | 0.134  (0.156) | | 0.107  (0.155) | | 0.136  (0.156) | | 0.160  (0.160) | | -0.135  (0.198) | | -0.125  (0.200) | | -0.122  (0.187) | | -0.084  (0.202) | |
| Volunteering *_2010_* | | -0.131  (0.135) | | 0.015  (0.047) | | 0.008  (0.045) | | 0.013  (0.047) | | -0.236  (0.157) | | 0.007  (0.065) | | 0.013  (0.065) | | 0.035  (0.063) | | -0.494*  (0.239) | | -0.566*  (0.253) | | -0.504*  (0.241) | | -0.466  (0.240) | |
| Volunteering² *_2010_* | | 0.064  (0.055) | | – | | – | | – | | 0.114  (0.060) | | – | | – | | – | | 0.199*  (0.093) | | 0.222*  (0.097) | | 0.202*  (0.094) | | 0.194*  (0.093) | |
| Age 65–75 | | | | | | | | | | | | | | | | | | | | | | | | |  |
| Lagged  outcome *_2010_* | | 0.846**  (0.035) | | 0.827**  (0.040) | | 0.840**  (0.035) | | 0.847**  (0.035) | | 0.639**  (0.041) | | 0.623**  (0.043) | | 0.629**  (0.042) | | 0.639**  (0.041) | | 1.386**  (0.132) | | 1.413**  (0.149) | | 1.384**  (0.134) | | 1.389**  (0.133) | |
| Membership *_2011_* | | -0.004  (0.171) | | -0.105  (0.169) | | -0.034  (0.170) | | -0.047  (0.173) | | 0.027  (0.209) | | -0.101  (0.211) | | -0.058  (0.209) | | 0.002  (0.211) | | 0.368  (0.298) | | 0.145  (0.324) | | 0.284  (0.298) | | 0.369  (0.307) | |
| Active participation *_2011_* | | 0.241  (0.201) | | 0.161  (0.207) | | 0.227  (0.198) | | 0.134  (0.211) | | 0.075  (0.224) | | 0.036  (0.223) | | 0.077  (0.217) | | 0.006  (0.232) | | 0.054  (0.342) | | -0.043  (0.365) | | -0.017  (0.344) | | -0.009  (0.351) | |
| Volunteering *_2010_* | | -0.041 (0.184) | | 0.037  (0.064) | | 0.034  (0.062) | | 0.023  (0.063) | | -0.043  (0.217) | | -0.042  (0.071) | | -0.013  (0.070) | | -0.019  (0.071) | | -0.384  (0.414) | | -0.024  (0.106) | | 0.035  (0.098) | | 0.038  (0.100) | |
| Volunteering² *_2010_* | | 0.031  (0.064) | | – | | – | | – | | 0.015  (0.079) | | – | | – | | – | | 0.157  (0.141) | | – | | – | | – | |

*Note.* Age groups were defined on the basis of participants’ age in 2010. Cells represent unstandardized linear or ordered logit regression coefficients with standard errors in parentheses. Model 1 = without control variables. Model 2 = adjusted for sample origin, sex, highest educational attainment in 2011, occupational status in 2011, income in 2011, employment status in 2011, cohabiting with partner in 2011, and having underage children in the household in 2011. Model 3 = adjusted for subjective health in 2011 and emotional well-being in 2011. Model 4 = adjusted for church attendance in 2012.

* *p* < .05. ** *p* < .01.

*Separate Analyses by Sex: Effects of Membership, Active Participation, and Volunteering on Alcohol Consumption in Two-Wave Analyses in Women*

| Predictors | Alcohol consumption past 12 months *_2013_* | | | | | | | | Maximum ethanol consumption past 7 days *_2013_* | | | | | | | | Risk of binge drinking past 7 days *_2013_* | | | | | | | |  |
| --- | --- | --- | --- | --- | --- | --- | --- | --- | --- | --- | --- | --- | --- | --- | --- | --- | --- | --- | --- | --- | --- | --- | --- | --- | --- |
|  | Model 1 | | Model 2 | | Model 3 | | Model 4 | | Model 1 | | Model 2 | | Model 3 | | Model 4 | | Model 1 | | Model 2 | | Model 3 | | Model 4 | |  |
| Age 14­–29 | | | | | | | | | | | | | | | | | | | | | | | | |  |
| Lagged  outcome *_2010_* | | 0.593**  (0.046) | | 0.567**  (0.048) | | 0.586**  (0.047) | | 0.593**  (0.045) | | 0.273**  (0.038) | | 0.249**  (0.038) | | 0.270**  (0.038) | | 0.267**  (0.038) | | 0.360**  (0.060) | | 0.338**  (0.062) | | 0.360**  (0.060) | | 0.358**  (0.060) | |
| Membership *_2011_* | | 0.034  (0.143) | | -0.045  (0.153) | | 0.040  (0.143) | | 0.051  (0.144) | | 0.148  (0.224) | | 0.134  (0.241) | | 0.160  (0.223) | | 0.211  (0.227) | | 0.006  (0.214) | | -0.014  (0.238) | | 0.012  (0.213) | | 0.061  (0.216) | |
| Active participation *_2011_* | | -0.131  (0.157) | | -0.081  (0.161) | | -0.145  (0.155) | | -0.117  (0.156) | | -0.729**  (0.243) | | -0.597*  (0.253) | | -0.724**  (0.241) | | -0.666**  (0.242) | | -0.606**  (0.233) | | -0.505*  (0.253) | | -0.600*  (0.234) | | -0.559*  (0.231) | |
| Volunteering *_2010_* | | -0.003  (0.175) | | -0.045  (0.056) | | -0.047  (0.056) | | -0.042  (0.056) | | 0.406  (0.257) | | -0.028  (0.081) | | 0.001  (0.081) | | 0.014  (0.080) | | 0.198  (0.246) | | -0.134  (0.075) | | -0.098  (0.072) | | -0.085  (0.070) | |
| Volunteering² *_2010_* | | -0.019  (0.075) | | – | | – | | – | | -0.182  (0.107) | | – | | – | | – | | -0.133  (0.104) | | – | | – | | – | |
| Age 40­–50 | | | | | | | | | | | | | | | | | | | | | | | | |  |
| Lagged  outcome *_2010_* | | 0.841**  (0.022) | | 0.810**  (0.025) | | 0.833**  (0.023) | | 0.839**  (0.023) | | 0.566**  (0.031) | | 0.530**  (0.033) | | 0.556**  (0.031) | | 0.568**  (0.031) | | 0.950**  (0.066) | | 0.944**  (0.071) | | 0.940**  (0.067) | | 0.954**  (0.067) | |
| Membership *_2011_* | | 0.075  (0.092) | | -0.035  (0.093) | | 0.047  (0.091) | | 0.082  (0.093) | | 0.170  (0.140) | | -0.098  (0.144) | | 0.115  (0.141) | | 0.162  (0.142) | | 0.262  (0.167) | | -0.040  (0.174) | | 0.211  (0.170) | | 0.250  (0.168) | |
| Active participation *_2011_* | | 0.021  (0.099) | | 0.011  (0.101) | | 0.026  (0.097) | | 0.043  (0.102) | | 0.116  (0.146) | | 0.032  (0.147) | | 0.110  (0.146) | | 0.105  (0.152) | | 0.072  (0.166) | | -0.023  (0.171) | | 0.086  (0.166) | | 0.067  (0.170) | |
| Volunteering *_2010_* | | 0.137  (0.104) | | 0.061  (0.036) | | 0.058  (0.035) | | 0.060  (0.035) | | 0.111  (0.166) | | 0.044  (0.054) | | 0.039  (0.053) | | 0.033  (0.054) | | 0.216  (0.188) | | 0.048  (0.067) | | 0.039  (0.061) | | 0.035  (0.062) | |
| Volunteering² *_2010_* | | -0.034  (0.042) | | – | | – | | – | | -0.031  (0.066) | | – | | – | | – | | -0.073  (0.072) | | – | | – | | – | |
| Age 65–75 | | | | | | | | | | | | | | | | | | | | | | | | |  |
| Lagged  outcome *_2010_* | | 0.775**  (0.037) | | 0.749**  (0.041) | | 0.761**  (0.038) | | 0.770**  (0.037) | | 0.593**  (0.039) | | 0.565**  (0.041) | | 0.585**  (0.039) | | 0.596**  (0.038) | | 1.223**  (0.122) | | 1.230**  (0.133) | | 1.222**  (0.127) | | 1.237**  (0.121) | |
| Membership *_2011_* | | -0.204  (0.169) | | -0.291  (0.173) | | -0.215  (0.168) | | -0.214  (0.175) | | 0.139  (0.189) | | 0.074  (0.188) | | 0.147  (0.185) | | 0.134  (0.190) | | 0.293  (0.303) | | 0.219  (0.309) | | 0.318  (0.302) | | 0.280  (0.299) | |
| Active participation *_2011_* | | 0.261  (0.174) | | 0.259  (0.177) | | 0.229  (0.173) | | 0.258  (0.176) | | -0.012  (0.185) | | 0.060  (0.186) | | -0.024  (0.182) | | -0.005  (0.192) | | 0.094  (0.295) | | 0.145  (0.316) | | 0.087  (0.299) | | 0.068  (0.298) | |
| Volunteering *_2010_* | | -0.104  (0.149) | | 0.051  (0.058) | | 0.047  (0.055) | | 0.066  (0.056) | | 0.161  (0.196) | | 0.030  (0.060) | | 0.046  (0.059) | | 0.064  (0.059) | | 0.136  (0.278) | | -0.042  (0.102) | | 0.003  (0.093) | | 0.012  (0.090) | |
| Volunteering² *_2010_* | | 0.068  (0.057) | | – | | – | | – | | -0.040  (0.077) | | – | | – | | – | | -0.051  (0.113) | | – | | – | | – | |

*Note.* Age groups were defined on the basis of participants’ age in 2010. Cells represent unstandardized linear or ordered logit regression coefficients with standard errors in parentheses. Model 1 = without control variables. Model 2 = adjusted for sample origin, sex, highest educational attainment in 2011, occupational status in 2011, income in 2011, employment status in 2011, cohabiting with partner in 2011, and having underage children in the household in 2011. Model 3 = adjusted for subjective health in 2011 and emotional well-being in 2011. Model 4 = adjusted for church attendance in 2012.

* *p* < .05. ** *p* < .01.

**Appendix D**

*Effects of Membership and Active Participation in Different Types of Voluntary Organizations on Pub Attendance and Smoking*

| Predictors | Pub attendance *_t_* | | | Smoker *_t_* | | | Smoking intensity *_t_* | | |
| --- | --- | --- | --- | --- | --- | --- | --- | --- | --- |
|  | Model 1 | Model 2 | Model 3 | Model 1 | Model 2 | Model 3 | Model 1 | Model 2 | Model 3 |
|  | Age 14–29 | | | | | | | | |
| *Within* |  |  |  |  |  |  |  |  |  |
| Membership in political party *_t-1_* | 0.250  [-0.098, 0.604] | 0.277  [-0.098, 0.627] | 0.253  [-0.111, 0.615] | 0.077  [-0.322, 0.450] | 0.109  [-0.371, 0.529] | 0.062  [-0.343, 0.503] | 0.099  [-0.345, 0.564] | 0.096  [-0.352, 0.535] | 0.100  [-0.337, 0.556] |
| Active in political party *_t-1_* | -0.085  [-0.505, 0.364] | -0.070  [-0.504, 0.393] | -0.051  [-0.474, 0.385] | 0.035  [-0.473, 0.515] | -0.082  [-0.572, 0.488] | -0.001  [-0.498, 0.557] | 0.197  [-0.388, 0.786] | 0.171  [-0.390, 0.760] | 0.222  [-0.357, 0.786] |
| Membership in service-orient. organization *_t-1_* | -0.041  [-0.157, 0.070] | -0.021  [-0.128, 0.089] | -0.046  [-0.159, 0.071] | -0.095  [-0.266, 0.074] | -0.101  [-0.252, 0.060] | -0.107  [-0.275, 0.073] | -0.066  [-0.264, 0.118] | -0.072  [-0.253, 0.111] | -0.057  [-0.260, 0.135] |
| Active in service-orient. organization *_t-1_* | -0.143**  [-0.244, -0.043] | -0.148**  [-0.250, -0.048] | -0.150**  [-0.252, -0.049] | -0.123  [-0.272, 0.024] | -0.106  [-0.257, 0.040] | -0.115  [-0.276, 0.033] | 0.033  [-0.164, 0.220] | 0.047  [-0.128, 0.231] | 0.033  [-0.145, 0.211] |
| Membership in mixed organization *_t-1_* | -0.042  [-0.104, 0.022] | -0.069*  [-0.126, -0.011] | -0.064*  [-0.122, -0.006] | 0.043  [-0.039, 0.111] | 0.029  [-0.046, 0.104] | 0.036  [-0.034, 0.109] | -0.019  [-0.096, 0.055] | -0.025  [-0.106, 0.058] | -0.012  [-0.094, 0.062] |
| Active in mixed organization *_t-1_* | -0.042  [-0.104, 0.022] | -0.020  [-0.085, 0.045] | -0.035  [-0.097, 0.030] | 0.049  [-0.045, 0.138] | 0.061  [-0.028, 0.163] | 0.054  [-0.036, 0.144] | 0.011  [-0.088, 0.119] | 0.031  [-0.075, 0.134] | 0.013  [-0.087, 0.115] |
| Age *_t_* | -0.066**  [-0.071, -0.061] | -0.043**  [-0.048, -0.038] | -0.066**  [-0.071, -0.061] | -0.054**  [-0.058, -0.050] | -0.049**  [-0.054, -0.045] | -0.055**  [-0.059, -0.051] | -0.003  [-0.008, 0.001] | -0.003  [-0.008, 0.001] | -0.004  [-0.008, 0.001] |
| *Between* |  |  |  |  |  |  |  |  |  |
| Ever member in political party | 0.109  [-0.241, 0.467] | -0.049  [-0.381, 0.277] | 0.138  [-0.222, 0.489] | -0.077  [-0.834, 0.675] | -0.079  [-0.788, 0.609] | -0.253  [-0.982, 0.462] | 0.034  [-0.494, 0.576] | 0.043  [-0.456, 0.555] | -0.034  [-0.556, 0.485] |
| Ever active in political party | 0.455*  [0.047, 0.878] | 0.142  [-0.231, 0.516] | 0.433*  [0.034, 0.849] | 0.424  [-0.456, 1.347] | 0.714  [-0.162, 1.584] | 0.498  [-0.363, 1.381] | 0.004  [-0.601, 0.595] | 0.253  [-0.338, 0.821] | 0.023  [-0.561, 0.615] |
| Ever member in service-orient. organization | -0.153  [-0.308, 0.005] | -0.174*  [-0.313, -0.034] | -0.152*  [-0.305, -0.003] | -1.178**  [-1.559, -0.815] | -1.057**  [-1.409, -0.728] | -1.192**  [-1.530, -0.857] | -0.053  [-0.318, 0.197] | 0.023  [-0.212, 0.273] | -0.067  [-0.311, 0.179] |
| Ever active in service-orient. organization | -0.293**  [-0.441, -0.144] | -0.240**  [-0.374, -0.110] | -0.287**  [-0.430, -0.140] | -0.227  [-0.587, 0.144] | -0.060  [-0.380, 0.272] | -0.219  [-0.535, 0.098] | -0.402**  [-0.642, -0.160] | -0.339**  [-0.564, -0.109] | -0.399**  [-0.627, -0.169] |
| Ever member in mixed organization | 0.271**  [0.180, 0.363] | 0.036  [-0.052, 0.122] | 0.237**  [0.141, 0.330] | -1.019**  [-1.237, -0.797] | -0.388**  [-0.597, -0.174] | -0.905**  [-1.116, -0.695] | -0.410**  [-0.563, -0.270] | -0.101  [-0.247, 0.046] | -0.373**  [-0.515, -0.235] |
| Ever active in mixed organization | -0.382**  [-0.481, -0.283] | -0.092*  [-0.180, -0.003] | -0.381**  [-0.479, -0.283] | -0.510**  [-0.741, -0.275] | -0.292**  [-0.519, -0.072] | -0.534**  [-0.748, -0.316] | -0.097  [-0.252, 0.064] | 0.002  [-0.157, 0.155] | -0.108  [-0.265, 0.045] |
|  | Age 40–50 | | | | | | | | |
| *Within* |  |  |  |  |  |  |  |  |  |
| Membership in political party *_t-1_* | -0.026  [-0.292, 0.213] | -0.046  [-0.277, 0.205] | -0.050  [-0.295, 0.200] | -0.031  [-0.423, 0.349] | -0.033  [-0.405, 0.322] | -0.076  [-0.438, 0.257] | -0.156  [-0.549, 0.247] | -0.164  [-0.561, 0.230] | -0.145  [-0.539, 0.241] |
| Active in political party *_t-1_* | 0.027  [-0.256, 0.311] | -0.008  [-0.285, 0.170] | 0.019  [-0.284, 0.297] | -0.413  [-0.872, 0.075] | -0.403  [-0.906, 0.170] | -0.390  [-0.858, 0.109] | -0.126  [-0.660, 0.398] | -0.093  [-0.598, 0.412] | -0.133  [-0.636, 0.383] |
| Membership in service-orient. organization *_t-1_* | -0.036  [-0.142, 0.066] | -0.033  [-0.142, 0.084] | -0.045  [-0.153, 0.067] | -0.196  [-0.403, 0.009] | -0.197  [-0.417, 0.063] | -0.189  [-0.443, 0.011] | -0.166  [-0.404, 0.067] | -0.169  [-0.428, 0.074] | -0.178  [-0.413, 0.069] |
| Active in service-orient. organization *_t-1_* | 0.049  [-0.059, 0.150] | 0.058  [-0.043, 0.162] | 0.047  [-0.059, 0.148] | 0.111  [-0.076, 0.339] | 0.139  [-0.102, 0.334] | 0.114  [-0.094, 0.368] | 0.088  [-0.145, 0.317] | 0.087  [-0.149, 0.325] | 0.101  [-0.131, 0.341] |
| Membership in mixed organization *_t-1_* | 0.056  [-0.012, 0.120] | 0.022  [-0.046, 0.089] | 0.046  [-0.029, 0.109] | -0.011  [-0.137, 0.090] | -0.002  [-0.108, 0.126] | -0.014  [-0.118, 0.106] | 0.149*  [0.032, 0.260] | 0.137*  [0.014, 0.258] | 0.154*  [0.037, 0.276] |
| Active in mixed organization *_t-1_* | -0.067*  [-0.141, -0.003] | -0.039  [-0.104, 0.026] | -0.066  [-0.131, 0.001] | -0.058  [-0.207, 0.091] | -0.055  [-00.183, 0.095] | -0.066  [-0.199, 0.060] | -0.075  [-0.214, 0.070] | -0.055  [-0.198, 0.092] | -0.072  [-0.216, 0.071] |
| Age *_t_* | -0.039**  [-0.045, -0.033] | -0.049**  [-0.055, -0.043] | -0.038**  [-0.044, -0.033] | -0.112**  [-0.120, -0.105] | -0.118**  [-0.126, -0.109] | -0.110**  [-0.117, -0.103] | -0.054**  [-0.060, -0.047] | -0.058**  [-0.065, -0.050] | -0.053**  [-0.059, -0.047] |
| *Between* |  |  |  |  |  |  |  |  |  |
| Ever member in political party | 0.689**  [0.322, 1.046] | 0.484**  [0.131, 0.835] | 0.698**  [0.347, 1.051] | 0.524  [-0.450, 1.479] | 0.588  [-0.319, 1.491] | 0.533  [-0.442, 1.488] | 0.244  [-0.386, 0.882] | 0.162  [-0.467, 0.782] | 0.261  [-0.365, 0.867] |
| Ever active in political party | -0.127  [-0.549, 0.285] | -0.160  [-0.569, 0.234] | -0.078  [-0.477, 0.330] | -0.463  [-1.536, 0.680] | -0.004  [-1.031, 1.073] | -0.269  [-1.372, 0.849] | -0.256  [-1.075, 0.531] | -0.232  [-1.035, 0.545] | -0.316  [-1.074, 0.480] |
| Ever member in service-orient. organization | -0.370**  [-0.601, -0.145] | -0.299**  [-0.516, -0.079] | -0.400**  [-0.630, -0.174] | -1.929**  [-2.697, -1.235] | -1.713**  [-2.401, -1.065] | -1.824**  [-2.549, -1.160] | -0.055  [-0.498, 0.387] | -0.045  [-0.513, 0.417] | -0.087  [-0.527, 0.364] |
| Ever active in service-orient. organization | -0.627**  [-0.855, -0.404] | -0.419**  [-0.634, -0.209] | -0.580**  [-0.798, -0.358] | -0.563  [-1.230, 0.077] | -0.459  [-1.174, 0.255] | -0.618  [-1.327, 0.086] | -0.466*  [-0.898, -0.046] | -0.365  [-0.817, 0.071] | -0.439*  [-0.872, -0.014] |
| Ever member in mixed organization | 0.463**  [0.306, 0.622] | 0.211*  [0.043, 0.377] | 0.377**  [0.219, 0.535] | -1.198**  [-1.646, -0.755] | -0.523*  [-0.968, -0.090] | -0.785**  [-1.215, -0.356] | -0.214  [-0.489, 0.062] | -0.135  [-0.433, 0.165] | -0.122  [-0.400, 0.158] |
| Ever active in mixed organization | 0.110  [-0.047, 0.271] | 0.084  [-0.067, 0.233] | 0.084  [-0.068, 0.244] | -1.173**  [-1.655, -0.697] | -0.931**  [-1.414, -0.437] | -1.140**  [-1.603, -0.689] | -0.327*  [-0.613, -0.043] | -0.300*  [-0.601, -0.012] | -0.327*  [-0.617, -0.050] |
|  | Age 65–75 | | | | | | | | |
| *Within* |  |  |  |  |  |  |  |  |  |
| Membership in political party *_t-1_* | 0.066  [-0.225, 0.345] | 0.051  [-0.220, 0.322] | 0.058  [-0.222, 0.334] | -0.577  [-1.300, 0.064] | -0.591  [-1.331, 0.087] | -0.618  [-1.495, 0.101] | 0.080  [-0.661, 0.828] | 0.289  [-0.495, 1.060] | 0.080  [-0.689, 0.816] |
| Active in political party *_t-1_* | 0.195  [-0.124, 0.514] | 0.190  [-0.114, 0.492] | 0.187  [-0.142, 0.493] | 0.014  [-0.935, 1.017] | 0.041  [-0.935, 0.921] | 0.074  [-0.792, 1.073] | -1.120  [-2.348, 0.040] | -0.810  [-2.123, 0.465] | -1.098  [-2.389, 0.054] |
| Membership in service-orient. organization *_t-1_* | -0.267**  [-0.435, -0.095] | -0.264**  [-0.430, -0.104] | -0.269**  [-0.439, -0.104] | -0.132  [-0.559, 0.264] | -0.151  [-0.572, 0.298] | -0.177  [-0.641, 0.215] | 0.025  [-0.429, 0.516] | 0.053  [-0.489, 0.573] | 0.024  [-0.475, 0.508] |
| Active in service-orient. organization *_t-1_* | 0.088  [-0.079, 0.249] | 0.071  [-0.082, 0.233] | 0.090  [-0.087, 0.247] | 0.000  [-0.373, 0.422] | 0.044  [-0.386, 0.453] | 0.058  [-0.378, 0.473] | 0.036  [-0.427, 0.475] | 0.094  [-0.436, 0.617] | -0.001  [-0.484, 0.539] |
| Membership in mixed organization *_t-1_* | 0.045  [-0.084, 0.175] | 0.023  [-0.101, 0.150] | 0.047  [-0.079, 0.178] | 0.034  [-0.242, 0.340] | 0.026  [-0.277, 0.325] | 0.040  [-0.228, 0.334] | -0.009  [-0.347, 0.321] | 0.059  [-0.303, 0.416] | -0.020  [-0.354, 0.271] |
| Active in mixed organization *_t-1_* | 0.064  [-0.068, 0.196] | 0.091  [-0.041, 0.223] | 0.062  [-0.060, 0.186] | -0.146  [-0.488, 0.194] | -0.095  [-0.455, 0.260] | -0.128  [-0.495, 0.165] | -0.042  [-0.428, 0.315] | -0.049  [-0.443, 0.348] | -0.062  [-0.406, 0.321] |
| Age *_t_* | -0.073**  [-0.084, -0.063] | -0.070**  [-0.081, -0.058] | -0.072**  [-0.083, -0.062] | -0.149**  [-0.167, -0.132] | -0.154**  [-0.174, -0.134] | -0.143**  [-0.162, -0.125] | -0.063**  [-0.082, -0.043] | -0.051**  [-0.080, -0.023] | -0.057**  [-0.077, -0.039] |
| *Between* |  |  |  |  |  |  |  |  |  |
| Ever member in political party | 0.369  [-0.097, 0.828] | 0.247  [-0.181, 0.686] | 0.339  [-0.122, 0.791] | -0.033  [-1.515, 1.672] | -0.060  [-1.779, 1.544] | 0.157  [-1.123, 1.631] | 0.261  [-0.757, 1.316] | 0.628  [-0.702, 2.063] | 0.329  [-0.716, 1.364] |
| Ever active in political party | 0.251  [-0.306, 0.815] | 0.077  [-0.465, 0.614] | 0.230  [-0.327, 0.800] | -0.674  [-2.789, 1.301] | -0.128  [-2.128, 1.930] | -0.603  [-2.383, 1.143] | 0.785  [-0.704, 2.357] | -0.982  [-2.890, 0.891] | 0.781  [-0.714, 2.319] |
| Ever member in service-orient. organization | -0.249  [-0.629, 0.130] | 0.111  [-0.297, 0.529] | -0.247  [-0.617, 0.125] | -1.359*  [-2.674, -0.028] | -1.110  [-2.677, 0.344] | -1.269  [-2.454, 0.025] | -0.113  [-0.982, 0.749] | -0.447  [-1.938, 0.941] | -0.050  [-0.964, 0.786] |
| Ever active in service-orient. organization | -0.679**  [-1.058, -0.300] | -0.622**  [-1.004, -0.246] | -0.766**  [-1.147, -0.388] | -2.157**  [-3.427, -0.846] | -2.153**  [-3.559, -0.781] | -1.716**  [-3.032, -0.592] | -0.874  [-1.745, 0.024] | -0.451  [-1.734, 0.877] | -0.868  [-1.755, 0.040] |
| Ever member in mixed organization | 0.624**  [0.337, 0.933] | 0.243  [-0.045, 0.521] | 0.554**  [0.274, 0.825] | -1.031*  [-2.034, -0.019] | -1.023  [-2.121, 0.003] | -0.860  [-1.756, 0.097] | -0.359  [-1.005, 0.259] | -1.194*  [-2.609, -0.093] | -0.350  [-0.996, 0.292] |
| Ever active in mixed organization | -0.212  [-0.517, 0.092] | 0.106  [-0.189, 0.397] | -0.246  [-0.532, 0.041] | -0.611  [-1.640, 0.437] | -0.800  [-1.920, 0.299] | -0.519  [-1.479, 0.412] | -0.392  [-1.069, 0.246] | -0.690  [-1.914, 0.389] | -0.365  [-1.062, 0.292] |

*Note.* Age groups were defined on the basis of participants’ age in 1996 for pub attendance and in 1992 for smoking. Cells represent unstandardized probit regression coefficients with Bayesian credibility intervals in square brackets. Model 1 = without control variables. Model 2 = adjusted for employment status, income, cohabiting with partner, and having underage children in the household at the within level and for sample origin, sex, average educational attainment, average occupational status, and average income at the between level. Model 3 = adjusted for subjective health and emotional well-being at both levels.

* *p* < .05. ** *p* < .01.

*Effects of Membership and Active Participation in Different Types of Voluntary Organizations on Alcohol Consumption in Two-Wave Analyses*

| Predictors | Alcohol consumption past 12 months *_2013_* | | | | Maximum ethanol consumption past 7 days *_2013_* | | | | Risk of binge drinking past 7 days *_2013_* | | | |
| --- | --- | --- | --- | --- | --- | --- | --- | --- | --- | --- | --- | --- |
|  | Model 1 | Model 2 | Model 3 | Model 4 | Model 1 | Model 2 | Model 3 | Model 4 | Model 1 | Model 2 | Model 3 | Model 4 |
|  | Age 14–29 | | | | | | | | | | | |
| Lagged  outcome *_2010_* | 0.607**  (0.032) | 0.583**  (0.033) | 0.604**  (0.032) | 0.607**  (0.032) | 0.299**  (0.028) | 0.265**  (0.029) | 0.298**  (0.028) | 0.295**  (0.028) | 0.392**  (0.045) | 0.381**  (0.047) | 0.391**  (0.046) | 0.388**  (0.045) |
| Membership in political party *_2011_* | -0.113  (0.475) | -0.200  (0.472) | -0.166  (0.480) | -0.100  (0.467) | -0.649  (0.570) | -0.740  (0.585) | -0.671  (0.566) | -0.612  (0.538) | -0.647  (0.691) | -0.684  (0.856) | -0.664  (0.630) | -0.644  (0.639) |
| Active in political party *_2011_* | 1.490**  (0.580) | 1.344*  (0.539) | 1.498*  (0.593) | 1.471**  (0.574) | 1.898**  (0.694) | 1.727*  (0.698) | 1.943**  (0.703) | 1.838**  (0.689) | 1.542  (0.879) | 1.397  (1.150) | 1.577*  (0.653) | 1.496*  (0.661) |
| Membership in service-orient. organization *_2011_* | 0.187  (0.170) | 0.208  (0.174) | 0.217  (0.171) | 0.214  (0.180) | 0.102  (0.283) | 0.135  (0.279) | 0.098  (0.282) | 0.214  (0.285) | 0.039  (0.249) | 0.080  (0.254) | 0.039  (0.249) | 0.139  (0.254) |
| Active in service-orient. organization *_2011_* | -0.179  (0.171) | -0.180  (0.170) | -0.200  (0.167) | -0.171  (0.170) | -0.534*  (0.269) | -0.477  (0.268) | -0.513  (0.265) | -0.447  (0.262) | -0.488*  (0.235) | -0.450  (0.239) | -0.481*  (0.232) | -0.429  (0.231) |
| Membership in mixed organization *_2011_* | -0.111  (0.121) | -0.179  (0.121) | -0.128  (0.121) | -0.111  (0.120) | -0.058  (0.186) | -0.109  (0.188) | -0.062  (0.186) | -0.043  (0.184) | 0.080  (0.155) | 0.085  (0.166) | 0.076  (0.156) | 0.086  (0.154) |
| Active in mixed organization *_2011_* | 0.160  (0.137) | 0.215  (0.133) | 0.152  (0.137) | 0.156  (0.137) | 0.061  (0.239) | 0.158  (0.228) | 0.051  (0.239) | 0.034  (0.238) | -0.032  (0.202) | 0.039  (0.207) | -0.036  (0.203) | -0.047  (0.202) |
| Volunteering *_2010_* | -0.028  (0.139) | -0.049  (0.046) | -0.054  (0.045) | -0.052  (0.046) | 0.213  (0.190) | -0.018  (0.067) | 0.000  (0.067) | 0.014  (0.068) | -0.013  (0.169) | -0.098  (0.058) | -0.079  (0.057) | -0.066  (0.058) |
| Volunteering² *_2010_* | -0.011  (0.058) | – | – | – | -0.093  (0.079) | – | – | – | -0.029  (0.069) | – | – | – |
|  | Age 40–50 | | | | | | | | | | | |
| Lagged  outcome *_2010_* | 0.833**  (0.017) | 0.806**  (0.019) | 0.828**  (0.017) | 0.833**  (0.017) | 0.580**  (0.023) | 0.539**  (0.025) | 0.571**  (0.024) | 0.581**  (0.023) | 0.918**  (0.048) | 0.911**  (0.050) | 0.913**  (0.049) | 0.920**  (0.048) |
| Membership in political party *_2011_* | -0.052  (0.323) | -0.013  (0.341) | -0.005  (0.326) | -0.051  (0.319) | 0.609*  (0.263) | 0.663*  (0.302) | 0.696**  (0.269) | 0.615*  (0.257) | 0.473  (0.472) | 0.547  (0.526) | 0.537  (0.466) | 0.476  (0.470) |
| Active in political party *_2011_* | -0.007  (0.330) | 0.039  (0.309) | -0.010  (0.348) | -0.015  (0.321) | 0.164  (0.469) | 0.173  (0.484) | 0.146  (0.489) | 0.160  (0.454) | -0.328  (0.558) | -0.242  (0.644) | -0.301  (0.570) | -0.341  (0.555) |
| Membership in service-orient. organization *_2011_* | -0.004  (0.139) | -0.003  (0.141) | -0.009  (0.139) | -0.021  (0.144) | 0.058  (0.193) | 0.046  (0.198) | 0.043  (0.194) | -0.010  (0.202) | 0.041  (0.220) | -0.028  (0.227) | 0.036  (0.221) | 0.004  (0.233) |
| Active in service-orient. organization *_2011_* | 0.006  (0.136) | -0.002  (0.134) | 0.003  (0.135) | -0.002  (0.134) | -0.253  (0.185) | -0.270  (0.184) | -0.264  (0.184) | -0.291  (0.185) | -0.152  (0.214) | -0.162  (0.216) | -0.162  (0.213) | -0.167  (0.214) |
| Membership in mixed organization *_2011_* | 0.116  (0.067) | 0.027  (0.069) | 0.095  (0.067) | 0.116  (0.067) | 0.169  (0.097) | -0.043  (0.099) | 0.125  (0.097) | 0.166  (0.096) | 0.261*  (0.113) | 0.055  (0.120) | 0.224  (0.115) | 0.260*  (0.113) |
| Active in mixed organization *_2011_* | -0.006  (0.073) | -0.020  (0.074) | -0.012  (0.073) | -0.006  (0.073) | 0.207  (0.109) | 0.153  (0.108) | 0.195  (0.109) | 0.203  (0.109) | 0.045  (0.132) | -0.010  (0.137) | 0.042  (0.132) | 0.042  (0.133) |
| Volunteering *_2010_* | 0.027  (0.085) | 0.044  (0.030) | 0.040  (0.030) | 0.045  (0.031) | 0.020  (0.124) | 0.047  (0.042) | 0.050  (0.042) | 0.055  (0.042) | -0.006  (0.148) | 0.028  (0.051) | 0.032  (0.049) | 0.036  (0.049) |
| Volunteering² *_2010_* | 0.007  (0.034) | – | – | – | 0.015  (0.050) | – | – | – | 0.018  (0.057) | – | – | – |
|  | Age 65–75 | | | | | | | | | | | |
| Lagged  outcome *_2010_* | 0.817**  (0.025) | 0.781**  (0.030) | 0.810**  (0.026) | 0.817**  (0.025) | 0.634**  (0.026) | 0.589**  (0.029) | 0.624**  (0.026) | 0.634**  (0.026) | 1.313**  (0.096) | 1.312**  (0.100) | 1.307**  (0.097) | 1.311**  (0.096) |
| Membership in political party *_2011_* | 0.320  (0.240) | 0.332  (0.246) | 0.299  (0.251) | 0.315  (0.249) | 0.181  (0.413) | 0.204  (0.386) | 0.159  (0.409) | 0.181  (0.414) | 0.551  (0.615) | 0.573  (0.622) | 0.533  (0.610) | 0.561  (0.611) |
| Active in political party *_2011_* | 0.070  (0.287) | -0.031  (0.300) | 0.030  (0.289) | 0.022  (0.289) | 0.291  (0.590) | 0.145  (0.551) | 0.306  (0.577) | 0.267  (0.589) | -0.614  (1.007) | -0.933  (1.058) | -0.649  (0.959) | -0.675  (0.989) |
| Membership in service-orient. organization *_2011_* | -0.058  (0.161) | -0.102  (0.165) | -0.083  (0.161) | -0.089  (0.174) | -0.002  (0.168) | -0.009  (0.171) | -0.033  (0.168) | 0.018  (0.177) | 0.251  (0.275) | 0.183  (0.287) | 0.183  (0.280) | 0.264  (0.290) |
| Active in service-orient. organization *_2011_* | 0.220  (0.175) | 0.196  (0.171) | 0.194  (0.173) | 0.172  (0.179) | 0.288  (0.177) | 0.300  (0.172) | 0.273  (0.172) | 0.294  (0.183) | 0.231  (0.299) | 0.242  (0.315) | 0.234  (0.299) | 0.232  (0.310) |
| Membership in mixed organization *_2011_* | -0.048  (0.133) | -0.136  (0.133) | -0.057  (0.134) | -0.056  (0.135) | 0.122  (0.141) | -0.020  (0.141) | 0.098  (0.141) | 0.118  (0.141) | 0.171  (0.213) | -0.030  (0.222) | 0.156  (0.213) | 0.156  (0.213) |
| Active in mixed organization *_2011_* | -0.008  (0.147) | -0.001  (0.150) | -0.026  (0.148) | -0.015  (0.148) | -0.259  (0.153) | -0.212  (0.153) | -0.255  (0.152) | -0.255  (0.153) | 0.006  (0.229) | -0.013  (0.242) | -0.003  (0.228) | 0.008  (0.229) |
| Volunteering *_2010_* | -0.134  (0.122) | 0.040  (0.042) | 0.034  (0.040) | 0.042  (0.040) | -0.003  (0.152) | -0.014  (0.048) | -0.003  (0.048) | 0.006  (0.048) | -0.169  (0.231) | -0.037  (0.074) | -0.015  (0.074) | -0.013  (0.074) |
| Volunteering² *_2010_* | 0.069  (0.045) | – | – | – | 0.003  (0.058) | – | – | – | 0.061  (0.085) | – | – | – |

*Note.* Age groups were defined on the basis of participants’ age in 2010. Cells represent unstandardized linear or ordered logit regression coefficients with standard errors in parentheses. Model 1 = without control variables. Model 2 = adjusted for sample origin, sex, highest educational attainment in 2011, occupational status in 2011, income in 2011, employment status in 2011, cohabiting with partner in 2011, and having underage children in the household in 2011. Model 3 = adjusted for subjective health in 2011 and emotional well-being in 2011. Model 4 = adjusted for church attendance in 2012.

* *p* < .05. ** *p* < .01.

**Appendix E**

To report effect sizes for probit regression coefficients, one needs to (a) calculate predicted probabilities from probit regression coefficients, and (b) use a suitable approach to compare calculated probabilities.

*The Calculation of Predicted Probabilities*

We referred to the Mplus User’s Guide where formulas for the calculation of predicted probabilities from probit regression coefficients are given.

In case of a binary outcome variable (e.g., binary indicator of smoking status), the probability of u given x can be expressed as:

P (u = 1 | x) = F (-t + b*x).

In case of an ordinal outcome variable with three categories (e.g., smoking intensity), the probability of u given x is:

P (u = 0 | x) = F (t_1_ - b*x),

P (u = 1 | x) = F (t_2_ - b*x) - F (t_1_ - b*x),

P (u = 2 | x) = F (-t_2_ + b*x).

The parameters in both formulas above represent:

F = the standard normal distribution function

b = the probit regression slope

t = the probit regression threshold

t_1_ = the first probit regression threshold in a model with an ordinal outcome variable

t_2_ = the second probit regression threshold in a model with an ordinal outcome variable.

As advised by Linda Muthén, one of the developers of MPlus, we did not use the parameters of a standard normal distribution (*M* = 0, *SD* = 1) to calculate predicted probabilities, but a corrected normal distribution that accounts for the variance of the latent response variable at the between level. The standard deviation of this distribution equals to

.

*The Comparison of Predicted Probabilities*

As effect size, we report the relative difference, in %, between two given values of x (with every other covariate fixed at zero, i.e., at its mean value or at its reference category for categorical predictors) in the predicted probability to belong to a given category of the outcome variable. This difference was calculated as follows:

% difference = P (u = 1 | x = 1 for binary predictors; x = *M* + 1*SD* for continuous predictors)/ P (u = 1 | x = 0 for binary predictors; x = *M* for continuous predictors)*100 – 100.
